# Pre-sowing grain treatment with bio-AgNPs stimulates plant growth and affects redox homeostasis in maize

**DOI:** 10.3389/fpls.2025.1494741

**Published:** 2025-05-22

**Authors:** Joanna Trzcińska-Wencel, Natalia Mucha, Mahendra Rai, Jarosław Tyburski, Patrycja Golińska

**Affiliations:** ^1^ Department of Microbiology, Faculty of Biological and Veterinary Sciences, Nicolaus Copernicus University in Toruń, Toruń, Poland; ^2^ Department of Plant Physiology and Biotechnology, Faculty of Biological and Veterinary Sciences, Nicolaus Copernicus University in Toruń, Toruń, Poland; ^3^ Nanobiotechnology Laboratory, Department of Biotechnology, Sant Gadge Baba Amravati University, Amravati, India; ^4^ Department of Chemistry, Federal University of Piaui (UFPI), Teresina, Piauí, Brazil

**Keywords:** biogenic nanoparticles, seed priming, crop protection, plant growth stimulators, *Zea mays*

## Abstract

**Introduction:**

In the pursuit of sustainable development, nanotechnology provides effective solutions for enhancing agricultural productivity. Nanomaterials (NMs) can be effective in increasing plant abiotic and biotic stress tolerance. Understanding the nanoparticles (NPs)–plant interaction is essential to identify the potential of NPs for growth stimulation and phytotoxicity risks. Therefore, this study aimed to evaluate the effects of biologically synthesized silver nanoparticles (AgNPs) from *Fusarium solani* IOR 825 on the growth of *Zea mays*. Furthermore, the effect of AgNPs on oxidative stress and the antioxidant response was assessed.

**Methods:**

AgNPs were efficiently synthesized from *F. solani* IOR 825 and characterized for physicochemical properties using transmission electron microscopy (TEM), nanoparticle tracking analysis (NTA), dynamic light scattering (DLS), X-ray diffraction (XRD), and Fourier transform infrared (FTIR) spectroscopy and measurement of Zeta potential. AgNPs at concentrations of 32, 128, and 512 µg mL^−1^ were used for the pre-sowing treatment of maize grains to inhibit microbial pathogens present on their surface. Sterilized maize grains were cultivated for 14 days for plantlet development. Subsequently, germination percentage (%G), mean germination time (MGT), germination rate index (GRI), fresh and dry weight (FW and DW), and the Ag content in plant organs and total chlorophyll content were analyzed. Hydrogen peroxide (H_2_O_2_) and malondialdehyde (MDA) were determined in leaves, roots, stems, and caryopses to assess the oxidative stress. The antioxidative system response to the AgNPs treatment was studied by determining total glutathione (GSH+GSSG) and ascorbate (ASC) contents as well as catalase (CAT), superoxide dismutase (SOD), peroxidase (POX), and ascorbate peroxidase (APX) activities.

**Results:**

AgNPs were spherical and small [TEM average diameter of 22.97 ± 9.4 nm, NTA average size of 43 ± 36 nm, and DLS average hydrodynamic diameters of 27.44 nm (14%) and 108.4 nm (86%)]. Zeta potential revealed that NPs were negatively charged [-19.5 mV (61.3%) and −2.93 mV (38.6%)]. The diffractogram of AgNPs confirmed the presence of a face-centered cubic structure of crystalline AgNPs, while FTIR spectra showed the presence of biomolecules on their surface. The results showed a dose-dependent effect on maize growth. The increase in length and fresh weight of plants treated with a AgNPs concentration of 512 µg mL^−1^ was noted. The treatment with all tested concentrations of AgNPs (32, 128, and 512 µg mL^−1^) resulted in increased dry weight of leaves. Reduced chlorophyll content was observed in plants treated with the highest tested concentration of AgNPs (512 µg mL^−1^). The treatment of grains with AgNPs decreased H_2_O_2_ levels in all organs, except the stem where the oxidant’s level increased. MDA levels were unaffected except for the highest tested concentration of AgNPs, which raised its content in leaves. ASC and total glutathione levels were increased in roots and caryopses, respectively. The highest impact of AgNPs treatment was determined for SOD activity, which decreased in leaves, stems, and caryopses and increased in roots. CAT activity was decreased in leaves, stems, and roots. There was a minor effect on POX and APX activities.

**Conclusion:**

The lowest tested concentration of AgNPs (32 µg mL^−1^) on maize efficiently inhibits maize-borne pathogens, without any negative effect on plant growth and chlorophyll content. Moreover, it does not provoke oxidative stress. However, AgNPs may affect cellular redox systems when their higher concentrations (128 and 512 µg mL^−1^) are used. The results indicate the potential use of biogenically synthesized AgNPs in agriculture through a crop-safe approach to eliminate pathogens and increase maize production efficiency.

## 1 Introduction

Maize (*Zea mays*), which belongs to the grass tribe Andropogoneae of the Gramineae (Poaceae) family, is one of the most important cereal crop plants, alongside wheat (*Triticum* spp.) and rice (*Oryza sativa*) (FAO, 2022). The main purposes of maize cultivation are grain acquisition (production of groats, flour, and starch), silage production (livestock feed), and the use of maize by-products for other purposes, such as biofuel or ethanol production (Rouf Shah et al., 2016). Maize-based food products play a crucial role as a source of nutrients (carbohydrates, proteins, fat, microelements, and macroelements) and a wide range of beneficial health substances such as vitamins (riboflavin, thiamine, and vitamins C and E) or xanthophylls (lutein and zeaxanthin) (Rouf Shah et al., 2016; Revilla et al., 2022). However, the crop yield of maize is affected by abiotic (water and nutrient availability or climate factors including low temperature) and biotic factors that can highly limit maize growth. Among the abiotic factors, low temperature inhibits the germination of maize grains and the growth of plants, particularly in the initial stage of seedling development (Zhang et al., 2020). The biotic factors are represented by a wide range of microbial pathogens, e.g., *Aspergillus flavus*, *Aspergillus parasiticus*, *Colletotrichum graminicola*, *Fusarium graminearum*, *Fusarium moniliforme*, *Penicillium citrinum*, and *Rhizopus stolonifera* (Goko et al., 2021; Oldenburg and Ellner, 2015). It should be emphasized that germination and early stages of seedling growth are highly sensitive to the presence of pathogenic microbes (Lamichhane et al., 2018).

To overcome the problems caused by the above-mentioned abiotic and biotic factors and enhance maize production efficiency, several techniques such as fertilization, pesticide application, intercropping, seed treatment, and genetically modified organism (GMO) cultivation are commonly used (FAO, 2024; Goodman, 2024). However, these techniques are not entirely effective or safe. For example, fertilization requires a high degree of precision; otherwise, it is ineffective, and excess fertilizer leaks into the environment, causing pollution (Srivastav et al., 2024). Moreover, chemical fertilizers cause soil degradation (e.g., acidification), which reduces nutrient availability and disrupts the soil microbial ecosystem, ultimately reducing soil productivity (Cao et al., 2025). In turn, the use of pesticides (e.g., organophosphates and carbamates) has a significant negative impact on human and animal health, as pesticide residues are detected in feed and food (EFSA (European Food Safety Authority), 2024). Pesticides also negatively affect the environment by reducing the population of beneficial soil microorganisms and weakening soil structure and fertility (Tripathi et al., 2020; Brunelle et al., 2024; Cao et al., 2025). Meanwhile, GMO crops are associated with limited human population trust and legal regulations (Goodman, 2024). Therefore, the development of agriculture is directed toward searching for new solutions and sustainable agricultural technologies, including the implementation of innovative methods for pre-sowing seed treatment to enhance germination efficiency and early development of plants (Biswas et al., 2023), thus improving crop yields.

Nanotechnology, particularly the use of nanoparticles (NPs), has the potential to contribute to the development of modern agriculture. NPs exhibit unique physical and chemical properties, including a high surface-to-volume ratio, and the ability to cross biological membranes and interact effectively with biological systems. In agriculture, NPs have the potential to be utilized as nano-pesticides, nano-fungicides, nano-herbicides, and nano-fertilizers, offering significant benefits. They can enhance nutrient uptake, improve stress resistance, and increase photosynthetic efficiency, resulting in improved growth and yield even under challenging conditions. Moreover, their use can reduce the environmental hazards of conventional agricultural chemicals (Singh et al., 2024; Wahab et al., 2024). NPs are effective even at low concentrations and can be delivered through various methods, including seed treatment, foliar spraying, and hydroponic delivery (Mawale et al., 2024; Nile et al., 2022). NPs, unlike traditional fertilizers, which often leach into groundwater and water reservoirs causing their pollution, provide control over time and efficient nutrient release for plants (Easwaran et al., 2024; Haydar et al., 2024). Nano-priming is a novel approach for the pre-sowing treatment of seeds with NPs to improve germination speed, promote seedlings’ vigor, and enhance plant tolerance to stress conditions (Cao et al., 2025; Zhao et al., 2024). Its effectiveness depends on the dose and physicochemical properties of the NPs used, the time and temperature of priming, seed viability, and many other factors (Abbasi Khalaki et al., 2021). Some studies have reported that nanomaterials can penetrate the seed coat, resulting in increased water absorption. This, in turn, stimulates the enzyme system, which leads to rapid germination and seedling development (Shang et al., 2019). Moreover, nano-enabled seed treatment can increase the disease resistance of crops by boosting immunity, which will reduce the use of pesticides. This unsophisticated, farmer-available, cost-effective, and environmentally friendly seed treatment approach may help crop plants fight climate change challenges (Cao et al., 2025; Zhao et al., 2024). Although the application of NPs in agriculture is still in its developmental stages, it holds promise for increasing crop production and resilience against various stressors (Su et al., 2019; Haydar et al., 2024). NPs for such applications can be synthesized using chemical, physical, and biological methods. Chemical and physical methods of NPs synthesis include techniques such as co-precipitation, the sol–gel method, and laser ablation. These procedures allow for the synthesis of uniform products with high efficiency but require the use of hazardous chemicals that pose health and environmental risks, high temperature, pressure, and energy, as well as additional post-processing steps such as purification or stabilization (Abid et al., 2022). The growing emphasis on environmental protection prompts scientists to invent eco-friendly methods of NPs synthesis that would reduce toxic pollutant formation and minimize harmful environmental impacts (Borehalli Mayegowda et al., 2023). Therefore, the use of plants or microorganisms is the preferred method for the synthesis of NPs, as it is simple, inexpensive, time-efficient, and environmentally friendly and has stable yields. Moreover, molecules from biological sources play a dual role as reducing and capping agents; the latter agent prevents NPs from agglomeration and makes them more bioavailable (Sidhu et al., 2022; Trzcińska-Wencel et al., 2023a). In addition, biological NPs are believed to be more biocompatible than those synthesized chemically (Dowlath et al., 2021; Xiong et al., 2022). To date, the methods of green synthesis include plant (Bernardo-Mazariegos et al., 2019; Masum et al., 2019), bacterial (Abdelgadir et al., 2024), fungal (Golinska et al., 2017; Trzcińska-Wencel et al., 2023b), or algal (Waqif et al., 2024) systems. Several studies have shown the great potential of fungus-mediated synthesis to provide high-yield, stable, and biologically active NPs. Fungal systems due to rapid growth, significant biomass production, secretion of enzymes, and adaptability to new conditions seem remarkably capable of synthesizing NPs, both intracellularly and extracellularly. The fungal-mediated synthesis of a wide range of NPs, including silver (AgNPs), gold (AuNPs), copper (CuNPs), and zinc (ZnONPs), has been explored among different genera such as *Aspergillus*, *Colletotrichum*, *Fusarium*, *Penicillium*, or *Trichoderma* (Rai and Golińska, 2023; Anjum et al., 2023). The physical, chemical, and biological properties of mycosynthesized NPs depend on the fungal strain, conditions of growth, preparation of fungal extract, and reaction conditions, e.g., type and concentration of precursor, time, pH, or temperature (Brady et al., 2023). Several studies have pointed out that biologically synthesized AgNPs have potential as antimicrobials or plant growth stimulators for application in agriculture (Mahakham et al., 2017; Acharya et al., 2020; Sencan et al., 2024). However, NPs–plant interactions depend on many variables, including the type, shape, and size of the NPs, and their dose and application method, as well as the plant species (Syu et al., 2014; Krishnasamy et al., 2024). AgNPs can activate the expression of genes related to cell proliferation, metabolism, and hormone signaling pathways (Syu et al., 2014). Recently, Koley et al. (2023) reported that AgNPs improve germination and increase plant biomass by affecting hydrolytic enzyme activity and modulating reactive oxygen species (ROS) generation in the seeds of chickpeas, peas, and mung beans. Other studies have demonstrated the dose-dependent effects of AgNPs treatment on seedling growth, biochemical parameters, and antioxidative system activity, highlighting the adverse effects of higher concentrations, implying a potential toxic effect (Karim et al., 2023). Since the results of studies on the effects of AgNPs on plants, including seed germination and subsequent seedling growth, are limited and inconclusive, there is still a need for further research (Li et al., 2017; Guilger-Casagrande et al., 2022).

The present work is a continuation of our study on efficient biosynthesis of AgNPs using *Fusarium solani* IOR 825 and their antimicrobial activity against bacterial and fungal plant pathogens, including the sterilization of maize grains (Trzcińska-Wencel et al., 2023b). Those preliminary studies also determined the positive effect of AgNPs on germination and basic growth parameters of 7-day-old maize seedlings. It was the first time that *F. solani* IOR 825 was used as an efficient, economical, harmless, eco-friendly, and acceptable method for the synthesis of small-sized, negatively charged, and stable (bio-capped) AgNPs. These NPs showed antibacterial and antifungal activities against a set of plant pathogens and effectively sterilized maize grains at low concentrations, preventing the development of grain-borne microorganisms. Based on these excellent outcomes, we aimed to develop AgNPs through the green chemistry route with the ambition that these particles contribute some beneficial effects to industrial agriculture in the future to protect and improve maize growth without posing toxic effects or accumulation in plants. Therefore, this study aims to evaluate the effect of three selected concentrations of AgNPs on the germination of maize grains and plantlet vigor, as well as on the oxidative stress parameters and the antioxidants response within individual plant organs, such as leaves, stems, roots, and caryopses of 14-day-old maize plantlets.

## 2 Materials and methods

### 2.1 Biosynthesis and physicochemical characteristics of AgNPs from *F. solani* IOR 825

The biosynthesis and characterization of AgNPs synthesized from *F. solani* IOR 825 were performed as described previously by Trzcińska-Wencel et al. (2023b). Briefly, AgNPs were synthesized using fungal autolysate in water. For this purpose, the fungal strain was grown in Potato Dextrose Broth (PDB, A&A Biotechnology, Gdańsk, Poland) at 26°C for 7 days, followed by centrifugation (6500 × *g*, 10 min), biomass washing with sterile distilled water, and resuspending in water (100 mL of water for 10 g of biomass) for 3 days for autolysis. Autolysate was centrifuged (4000 × *g*, 5 min), filtered by sterile filter paper, and used for challenging with 100 mM silver nitrate (AgNO_3_; final concentration of 1 mM). The reaction mixture was sunlight-treated and incubated for 7 days in darkness. After this incubation period, AgNPs were centrifuged (13000 × *g*, 1 hour) and dried at 37°C. The mass of AgNPs was determined in mg, and the powder was used to prepare the stock solution of 2048 µg mL^−1^ for further analyses. For maize grain treatment, the AgNPs stock solution was used to prepare final (32, 128, and 512 µg mL^−1^) concentrations (Trzcińska-Wencel et al., 2023b).

The biosynthesis of AgNPs was confirmed using UV–Vis spectroscopy (NanoDrop One, Thermo Fisher Scientific, Waltham, MA, USA) at the wavelength range 200–700 nm with a resolution of 1 nm. The size and shape of AgNPs were determined using transmission electron microscopy (TEM) (FEI, Tecnai 12 Netherland) after applying AgNPs solution on a carbon-coated copper grid with 400-μm mesh size and drying at room temperature for 24 hours. The size of AgNPs was measured based on TEM micrographs using the ImageJ software. X-ray diffraction (XRD) (X’ Pert PRO Analytical X6 diffractometer, PANalytical, Almelo, Netherlands) with Cu Kα (*λ* = 1.54056 Å) radiation source and Ni were used with a filter in the 2θ range 5°–120°. The functional groups on the surface of AgNPs were determined by Fourier transform infrared (FTIR) spectroscopy (Spectrum 2000, Perkin-Elmer, Waltham, MA, USA). Before analysis, AgNPs powder was combined with KBr (1:100 ratio, w/w) and analyzed in the range 400–4000 cm^−1^ at a resolution of 4 cm^−1^. The size distribution and surface potential of AgNPs in Milli-Q water solution were evaluated by nanoparticle tracking analysis (NTA LM20, NanoSight Limited, Amesbury, UK), dynamic light scattering (DLS), and Zeta potential measurement (Zetasizer Nano-ZS 90, Malvern, UK). The software provided by the equipment manufacturer was used to analyze the obtained results, namely, NTA, version 2.3 Build 0033, and Zetasizer Software, version 6.32.

### 2.2 Surface sterilization of maize grains and growth conditions

Maize (*Z. mays*) grains (Torseed S.A, Toruń, Poland) were sterilized in previously selected concentrations (32 and 128 µg mL^−1^) of AgNPs as described by Trzcińska-Wencel et al. (2023b), while a concentration of 512 µg mL^−1^ was added to this study to expand significantly the concentration range of AgNPs used for the treatment of grains. Briefly, for each variant, 25 grains were selected and sterilized at room temperature for 30 min with 25 mL of 30% hydrogen peroxide (H_2_O_2_) and 70% ethanol (1:1, v:v) or with 25 mL of AgNPs solutions at the concentrations of 32, 128, and 512 µg mL^−1^ and washed five times with sterile distilled water. Grains were placed on ½ Murashige and Skoog (MS) agar and germinated at 22°C ± 2°C for 14 days. The 14-day-old plantlets at the V2 growth stage were harvested, their length and fresh and dry weight were measured, and the plant material for biochemical analysis was frozen in liquid nitrogen immediately after harvesting and stored at −80°C. All these parameters were estimated for roots, leaves, stems, and caryopses separately (**Supplementary Figure S1**).

### 2.3 Maize grain germination and plantlet parameters

The parameters of grain germination were calculated as follows:


G%(%)=(∑n/N)×100

where G% is the germination percentage, Ʃn is the total number of grains germinated after 14 days, and N is the total number of grains sown (Scott et al., 1984).


MGT=Σ f∗x/Σn

where MGT is the mean germination time, f is the number of germinated grains at day x, x is the number of days from sowing, and Ʃn is the total number of germinated grains (Orchard, 1977).


GRI=G1/1+G2/2+…+Gx/x

where GRI is the germination rate index and G1, G2, …, Gx is the germination percentage in the subsequent days after sowing (Esechie, 1994).


Vigor index I =G% × PL

where G% is the germination percentage and PL is the length of plantlets.


Vigor index II=G% × PDW

where G% is the germination percentage and PDW is the dry weight of plantlets (Abdul-Baki and Anderson, 1973).

The length of the shoots and roots was measured using a ruler and expressed in cm; the fresh and dry weight of the leaves, stems, roots, and caryopses were determined in mg.

### 2.4 Detection of AgNPs in maize

The plant material was washed and dried at 50°C for 48 hours, then finely powdered, and used for energy dispersive spectroscopy (EDS) analysis to assess elemental composition using a scanning electron microscope (LEO Electron Microscopy model 1430 VP Ltd., UK) coupled with an energy dispersive X-ray spectrometer (Quantax 200 with XFlash 4010 detector, Bruker AXS, Karlsruhe, Germany) (Kumari et al., 2024).

### 2.5 Leaf chlorophyll content

The total chlorophyll content in maize leaves was assayed according to the method described by Witham et al. (1971). Shortly, the powdered leaf tissue (0.5 g) was extracted with 1.5 mL of 80% cooled acetone and then centrifuged at 5000 × *g* for 5 min (Thermo Fisher Scientific, USA). The supernatant was drained into a 15-mL test tube, and the extraction of the remaining pellet was repeated five times (until the green color disappeared). Supernatants were combined, and the absorbance of the samples was read at λ_645nm_ and λ_663nm_ using a U-1800 spectrophotometer (Hitachi, Tokyo, Japan). The amount of total chlorophyll was calculated based on the following formula:


$$\text{Total chlorophyll}\;(\text{mg per g FW})=20.2\times \left(\text{Abs }\lambda_{645\;\mathrm{nm}}\right)+8.02\times \left(\text{Abs }\lambda_{663\;\mathrm{nm}}\right)\times \left(\text{V}/1000\times \text{FW}\right)$$

where V is the final volume of the extract (mL) and FW is the fresh weight of the leaf (g).

### 2.6 Oxidative stress parameters and the antioxidant system activity

#### 2.6.1 Hydrogen peroxide content

H_2_O_2_ content was determined according to the method described by Veljovic-Jovanovic et al. (2002), with their own modifications. The plant material (0.5 g) was ground in liquid nitrogen with a mortar and pestle and extracted with 5 mL of 0.1% trichloroacetic acid (TCA). After centrifugation at 10000 × *g* at 4°C for 10 min (Thermo Fisher Scientific, USA), 750 µL of supernatant was taken for assay. The reaction mixture contained 125 µL of 19.8 mM 3-(dimethylamino)benzoic acid (DMAB) (Sigma, St. Louis, MO, USA) in 0.1 M phosphate buffer (pH 6.5) and 115 µL of 0.456 mM 3-methyl-2-benzothiazolinone hydrazone hydrochloride (MBTH) and 10 µL of horseradish peroxidase (HRP) (Sigma) (25 U in the final volume of 1 mL). After incubation at 25°C for 20 min, the absorbance was measured at λ_590nm_ using a U-1800 spectrophotometer (Hitachi, Tokyo, Japan), and H_2_O_2_ concentration (µmol per 1 g of fresh weight) was calculated from the standard curve.

#### 2.6.2 Lipid peroxidation by determination of malondialdehyde level

Lipid peroxidation was determined by assessing malondialdehyde (MDA) level after MDA–thiobarbituric acid (TBA) complex formation under acidic conditions (Hodges et al., 1999); 0.5 g of each plant organ was homogenized in liquid nitrogen and extracted with 5 mL of 80% ethanol supplemented with 0.01% butylated hydroxytoluene (BHT). The extract was centrifuged at 3000 × *g* and 4°C for 10 min (Thermo Fisher Scientific, USA). The supernatant was mixed with 20% TCA containing 0.5% TBA, heated at 95°C for 20 min in a water bath, and then cooled immediately on ice. The sample was centrifuged at 3000 × *g* and 4°C for 10 min. The absorbance of the supernatant was measured at wavelengths 600, 532, and 440 nm (U-1800 spectrophotometer, Hitachi, Tokyo, Japan). The MDA level was calculated using the following equations (Equations 1–3):


(1) $$\left[\left(\text{Abs}532_{+\text{TBA}}-\text{Abs}600_{+\text{TBA}}\right)-\left(\text{Abs}532_{-\text{TBA}}-\text{Abs}600_{-\text{TBA}}\right)\right])=\text{A}$$

(2) $$\left[\left(\text{Abs}440_{+\mathrm{TBA}}-\text{Abs}600_{+\mathrm{TBA}}\right)\times 0.0571\right])=\text{B}$$

(3) $$\text{MDA equiv }\left({\text{nmol mL}}^{-1}\right)=\left(\left(\text{A}-\text{B}\right)/\epsilon \right)\times 10^{3}$$

where ϵ is corrected extinction coefficient of MDA (157 mM^−1^ cm^−1^), Abs532_+TBA_ − Abs600_+TBA_ is the absorbance of TBA–MDA complexes at 532 nm corrected for non-specific absorbance at 600 nm, Abs532_−TBA_ − Abs600_-TBA_ is the absorbance of compounds in extract solution without TBA at 532 nm corrected for non-specific absorbance at 600 nm, and [(Abs440_+TBA_ − Abs600_+TBA_) × 0.0571] is the correction for non-specific TBA–sugar complexes according to Hodges et al. (1999).

#### 2.6.3 Total glutathione content

One milliliter of 5% 5-sulfosalicylic acid (SSA) was used for the deproteinization of 0.1 g of homogenized plant material. After 10-min incubation on ice, the samples were centrifuged at 10000 × *g* for 10 min. Then, the supernatant was 20-fold diluted and used for analysis. Total glutathione level was determined by assessing the reduction rate of 5,5′-dithiobis(2-nitrobenzoic acid) (DTNB) to yellow 5-thio-2-nitrobenzoic acid (TNB) by GSH with Glutathione Assay Kit (Catalog Number CS0260, Sigma-Aldrich, USA), according to the manufacturer’s instruction. In the reaction mixture, the glutathione reductase reduced the glutathione disulfide (GSSG) to GSH at the expense of NADPH oxidation. TNB formation rate was measured spectrophotometrically at λ_412nm_ for 5 min using a plate reader (SpectraMax iD3 Multi-Mode Microplate Reader, Molecular Devices, San Jose, CA, USA). The results were calculated by comparison to a standard curve based on a series of GSH concentrations and expressed as nmol of total glutathione (GSH+GSSG) per 1 g of FW.

#### 2.6.4 Reduced and total (reduced and oxidized) ascorbate contents and ascorbate redox ratio

The plant material (0.5 g) was ground in liquid nitrogen and mixed with 5 mL of 5% TCA. After 10 min of incubation on ice, the samples were centrifuged at 14000 × *g* at 4°C for 10 min (Thermo Fisher Scientific, USA), and the supernatant was used for analyses. The level of reduced ascorbate (ASC) in the extract was estimated by colorimetric determination of the α,α′-bipyridyl complex formed with ferrous ions (Fe^2+^), which were reduced from ferric ions (Fe^3+^) by ascorbate from plant extract. To determine ASC in samples, a reaction mixture was prepared by adding the following in sequential order: 135 µL of supernatant, 33.6 µL of deionized water, 40 µL of 85% H_3_PO_4_, 685 µL of 0.5% α,α′-bipyridyl, and 140 µL of 1% FeCl_3_. To determine the total pool of ascorbate, i.e., the ASC and dehydroascorbate (DHA), 16.8 µL of 10 mM dithiothreitol (DTT) and 16.8 µL of 80 mM K_2_HPO_4_ were added to 135 µL of extract and incubated 5 min at room temperature to ensure reduction of DHA to ASC. Then, the extract was mixed with 40 µL of 85% H_3_PO_4_, 685 µL of 0.5% α,α′-bipyridyl, and 140 µL of 1% FeCl_3_. After 30-min incubation at room temperature, all samples were centrifuged at 14000 × *g* for 5 min, and the absorbance of the supernatant was measured at λ_525nm_ (U-1800 spectrophotometer, Hitachi, Tokyo, Japan) and compared to the standard curve of ASC in the of range of 0–50 µg mL^−1^. Based on the measurement results, the ASC level and the total pool of ASC and DHA were calculated and presented as mg g FW^−1^. To determine the redox status, the proportion of the reduced form in the total pool was calculated and presented as ASC/ASC + DHA ratio.

### 2.7 Antioxidant enzyme activities

#### 2.7.1 Protein extraction

Frozen samples (0.5 g) were ground in liquid nitrogen and extracted with 2.25 mL of homogenization buffer composed of 50 mM phosphate buffer, pH 7.5, 2 mM EDTA, 8 mM MgCl_2_, 0.1% Triton X-100, and 4 mM DTT and centrifuged at 14000 × *g* at 4°C for 15 min (Thermo Fisher Scientific, USA). The extract for ascorbate peroxidase (APX) activity assay was prepared with 50 mM phosphate buffer, pH 7.5, 5 mM ascorbate, and 1 mM EDTA. Total protein was assayed according to Bradford (1976) with bovine serum albumin (BSA) in the concentration range of 50–400 µg mL^−1^ as the standard.

#### 2.7.2 Activity of catalase

The activity of catalase was assayed by continuously measuring the decrease of H_2_O_2_ concentration in the sample (Rao et al., 1996). The reaction mixture consisted of 1.5 µL of H_2_O_2_ (30%), 40–100 μL of plant extract (depending on the plant organ), and 100 mM phosphate buffer, pH 7.0, in a final volume of 1000 μL. The assay mixture was thoroughly mixed in a spectrophotometric quartz cuvette (1.5-mL volume). Then, the decrease in the λ_240nm_ was measured for 90 s with a U-1800 spectrophotometer (Hitachi, Tokyo, Japan). The results were processed to calculate the activity of catalase and presented as U per mg of protein.

#### 2.7.3 Activity of superoxide dismutase

Superoxide dismutase activity was assessed by measuring the inhibition of nitroblue tetrazolium (NBT) to formazan reduction by superoxide radical, as described by Beauchamp and Fridovich (1971). To perform the assay, 25 μL of enzyme extract was mixed with 75 μL of extraction buffer and 1.5 mL of reaction mixture (50 mM phosphate buffer, pH 7.8, 0.67 mM NBT, 1 mM l-methionine, 0.33 mM EDTA, and 0.0033 mM riboflavin). For the control assay, the enzyme extract was substituted by an extraction buffer, and for negative control, H_2_O was used instead of the reaction mixture. Samples were incubated for 10 min in light. After that, the absorbance at λ_560nm_ was measured using a U-1800 spectrophotometer (Hitachi, Tokyo, Japan). The unit (U) of SOD activity was defined as the amount of enzyme that caused 50% inhibition of the photochemical reduction of NBT to formazan constitutes, and the results were demonstrated as U of enzyme per mg of protein.

#### 2.7.4 Activity of peroxidase

Peroxidase activity was assessed by determining the rate of pyrogallol oxidation to purpurogallin, in the presence of H_2_O_2_, as described previously by Tyburski and Mucha (2023). The increase in absorbance at λ_420nm_ was followed in the reaction mixture composed of 100 mM phosphate buffer, pH 6.0, 60 mM pyrogallol, 0.66 mM H_2_O_2_, and 5–80 μL of plant extract (depending on plant organ) for 90 s with a U-1800 spectrophotometer (Hitachi, Tokyo, Japan). To correct the results for non-enzymatic oxidation of pyrogallol, enzyme-free assays were performed. Peroxidase activity was calculated using the millimolar extinction coefficient of purpurogallin, ϵ = 12 mM^−1^ cm^−1^, and expressed as μmol pyrogallol min^−1^ mg^−1^ of total protein.

#### 2.7.5 Activity of ascorbate peroxidase

The activity of ascorbate peroxidase was evaluated by measuring the rate of H_2_O_2_ decomposition in the reaction mixture composed of 970 µL of reaction buffer (50 mM phosphate buffer, pH 7.5, and 1 mM EDTA), 10 µL of 50 mM ascorbate, and 10 µL of enzyme extract. The reaction was initiated by adding 10 µL of 0.2 mM H_2_O_2_, and the decrease in absorbance at λ_290nm_ was performed over 90 s (Chen and Asada, 1989; Rao et al., 1996). APX activity was calculated using the molar extinction coefficient of 2.8 mM^−1^ cm^−1^ for ascorbate and presented as μmol ascorbate min^−1^ mg^−1^ of total protein.

### 2.8 Statistical analysis

The data analysis was performed using GraphPad Prism version 10.0.0 (GraphPad Software, Boston, MA, USA). The results were shown as a mean ± standard error (SE). The means were then compared to determine statistical significance (if *p* < 0.05) by one-way ANOVA and *post-hoc* Tukey’s test. The growth, oxidative stress, and antioxidant parameters quantified in the organs of plantlets developed from AgNPs-treated grains were used to perform principal component analysis (PCA) and hierarchical cluster analysis (HCA) with R 4.4.2 (R Foundation for Statistical Computing, Vienna, Austria) using the factoextra, ggplot2, and dendextend R packages (Galili, 2015; Kassambara and Mundt, 2020; Wickham, 2016).

## 3 Results

### 3.1 Biosynthesis and physicochemical characteristics of AgNPs from *F. solani* IOR 825

**Figure 1:**
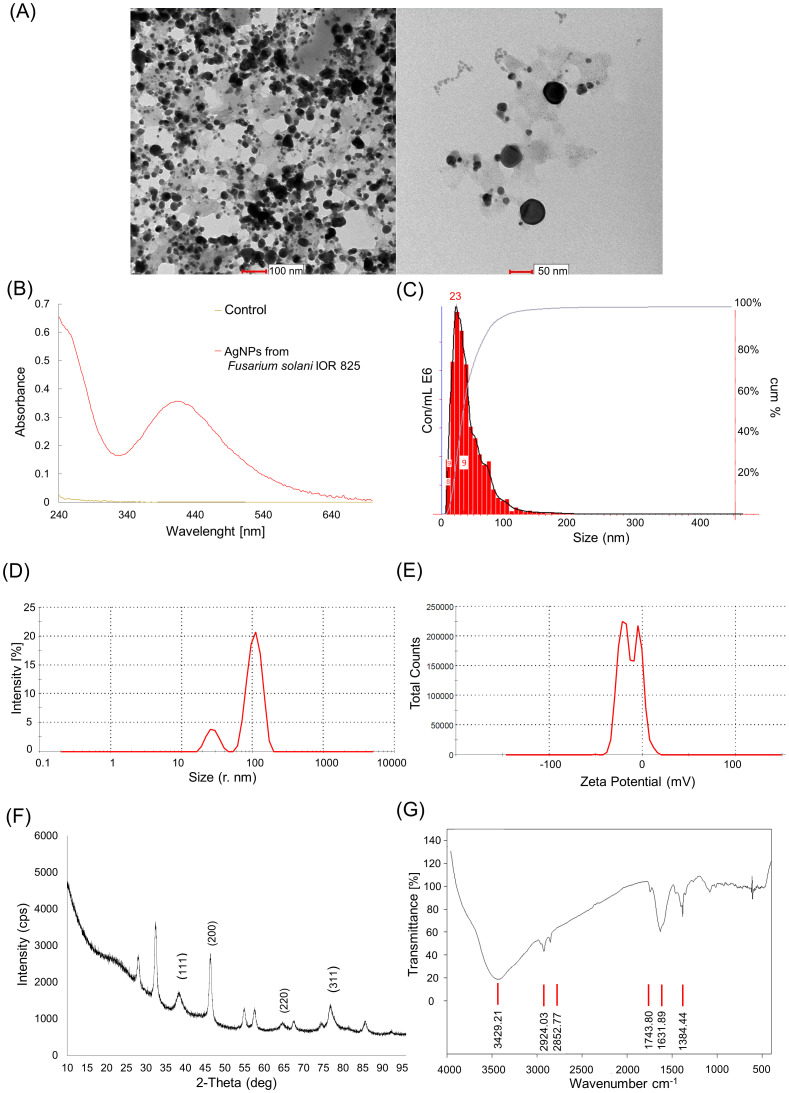
Detection and physicochemical characteristics of AgNPs synthesized from *Fusarium solani* IOR 825: transmission electron microscopy (TEM) micrographs **(A)**, UV–Vis spectrum **(B)**, size distribution from nanoparticle tracking analysis (NTA) **(C)**, size distribution from dynamic light scattering (DLS) analysis **(D)**, Zeta potential **(E)**, diffractogram from X-ray diffraction analysis **(F)**, and Fourier transform infrared (FTIR) spectrum **(G)**.

TEM analysis confirmed the formation of spherical AgNPs with sizes ranging from 8.9 to 47.9 nm and an average diameter of 22.97 ± 9.4 nm (**Figure 1A**). UV–visible absorption spectroscopic analysis of the mycosynthesized AgNPs showed a characteristic peak at 419 nm (**Figure 1B**). The results of nanoparticle tracking analysis (NTA) confirmed that the AgNPs exhibited an average size of 43 ± 36 nm and most frequently a diameter of 23 nm (**Figure 1C**). The results of DLS indicated that average hydrodynamic diameters of AgNPs were found to be 27.44 (14%) and 108.4 nm (86%), as shown in **Figure 1D**, while Zeta potential values were found to be −19.5 (61.3%) and −2.93 mV (38.6%) (**Figure 1E**). The diffractogram of AgNPs showed peaks at 38.63, 46.41, 65.10, and 77.09 corresponding to (1 1 1), (2 0 0), (2 2 0), and (3 1 1) planes of the face-centered cubic (fcc) silver crystal, respectively (**Figure 1F**). FTIR spectra showed absorption bands at 3429.21 cm^−1^ (N–H stretching, amines), 2924.03 cm^−1^ (C–H stretching, alkane), 2852.77 cm^−1^ (C–H stretching, alkane), 1743.8 cm^−1^ (C–H bending, aromatic compound), 1631.89 cm^−1^ (C=C stretching, alkene), and 1384.44 cm^−1^ (C–H bending, alkane) (**Figure 1G**).

### 3.2 Germination and growth parameters

**Figure 2:**
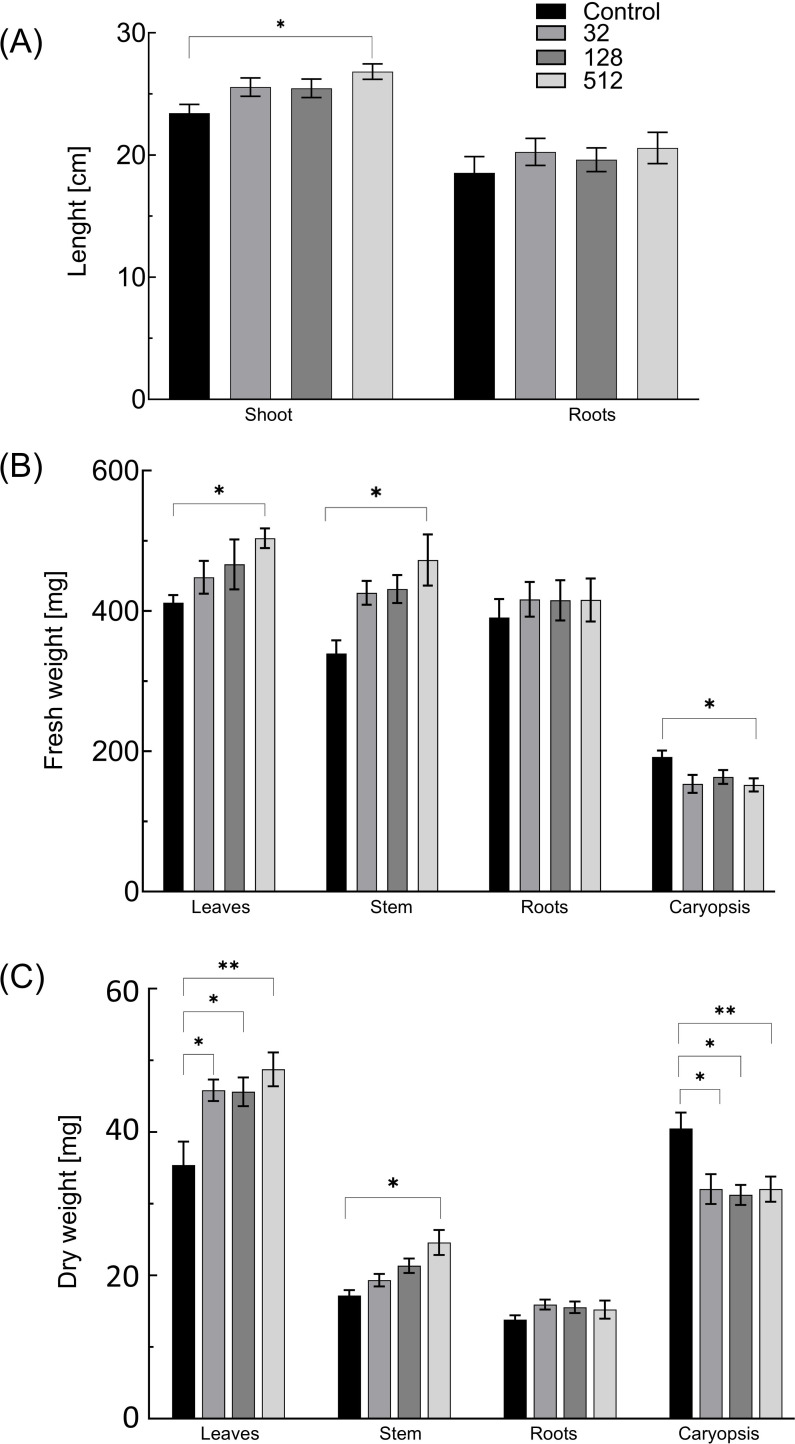
The length of shoots and roots **(A)** and fresh **(B)** and dry weight **(C)** of 14-day-old maize plantlets (n=30) after sterilization of grains with AgNPs. Data presented as mean and standard error (SE) and statistical significance (p-value: *p ≤ 0.05 and **p ≤ 0.01).

The surface sterilization of maize grains with AgNPs from *F. solani* IOR 825 at the concentration range tested did not affect the germination percentage (%G), mean germination time (MGT), and germination rate index (GRI), as shown in **Table 1**. The gradual improvement in plant growth was observed after treatment with increasing AgNPs concentrations, when compared to control, and reflected in the increase in shoot length, fresh weight of leaves, and stem of plants developed from AgNPs-treated grains (**Figure 2**; **Supplementary Table S1**). The strongest effect was observed for AgNPs treatment at a concentration of 512 µg mL^−1^, with improvements in shoot (13.3%) and root (11%) lengths, as well as fresh weight of leaves (22.3%) and stems (39.2%) and dry weight of leaves (37.8%) and stems (43.1%). This was also demonstrated in vigor indexes I and II, which increased to 4334.3 (I) and 8096.1 (II), compared to the controls, which were 3980.6 and 6259.7, respectively (**Table 1**). However, approximately 20% reduction in caryopsis weight was observed. Nevertheless, the stimulatory effect of the AgNPs treatment on root development was observed, and the difference with the untreated control did not pass the significance test.

**Table 1:** Germination parameters of maize grains after pre-treatment with AgNPs from *Fusarium solani* IOR 825.

AgNPs concentration (µg mL^−1^)	% Germination	Vigor index I	Vigor index II	MGT (days)	GRI (%/day)
0	94.3	3980.6	6259.7	3.2	31.0
32	95.7	4384.7	7759.7	3.2	31.4
128	92.9	4185.3	7660.5	3.2	31.0
512	91.4	4334.3*	8096.1*	3.2	30.5

MGT, mean germination time; GRI, germination rate index; AgNPs, silver nanoparticles.

^*^Statistical significance (*p*-value < 0.05) between AgNPs treatment and control (n=65).

### 3.3 AgNPs accumulation in maize

The analysis of elemental composition of plants developed from AgNPs-treated grains showed no significant difference in Ag content between tested and control samples, as shown in **Supplementary Table S2**.

### 3.4 Total chlorophyll content

**Figure 3:**
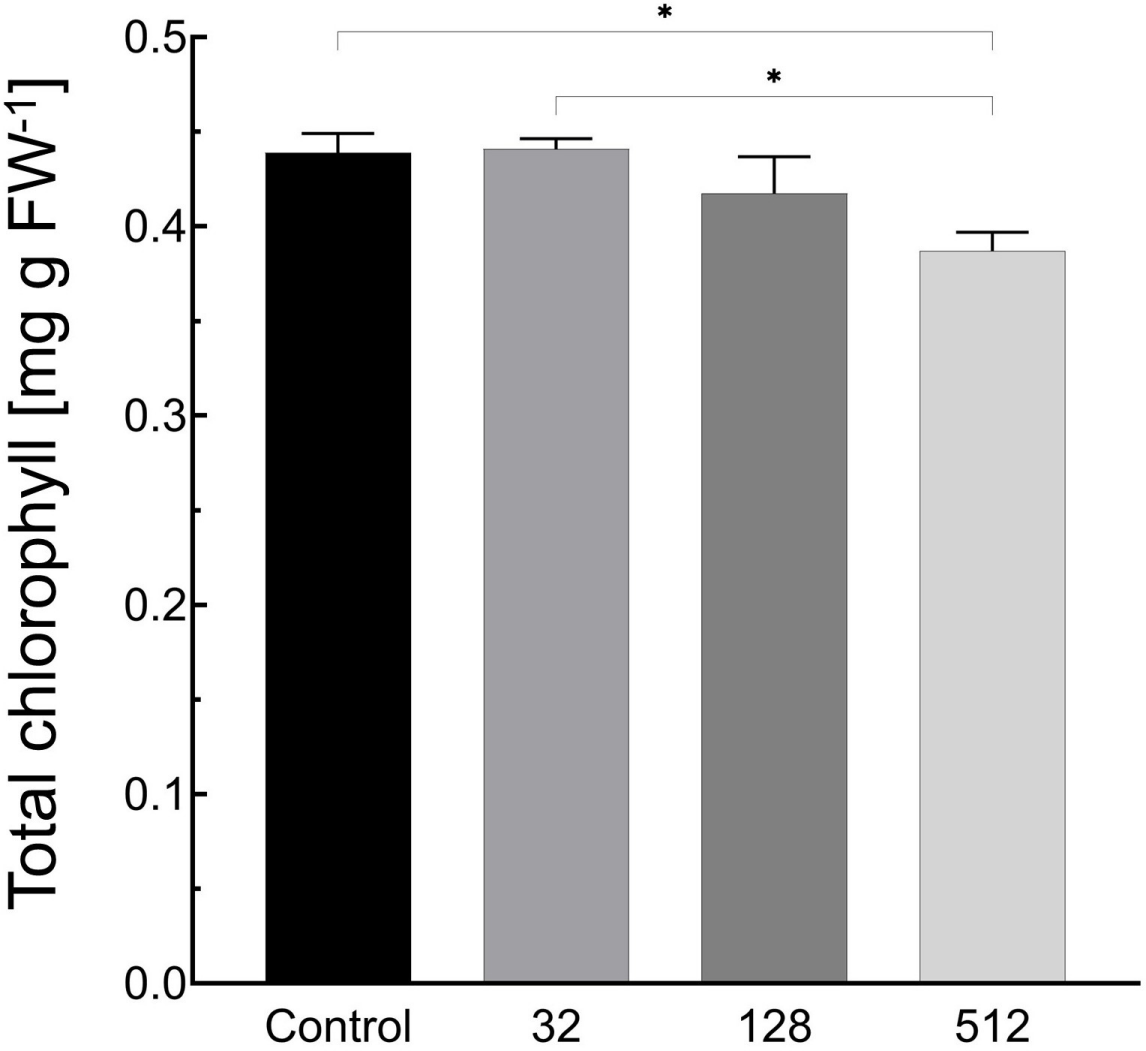
Influence of maize grain sterilization with AgNPs on the chlorophyll content in leaves of 14-day-old maize plantlets (n=9). Data presented as mean and standard error (± SE) and statistical significance (*p*-value: *p ≤ 0.05).

AgNPs treatments at concentrations of 32 and 128 µg mL^−1^ showed negligible effect on total chlorophyll content in the leaves of plantlets. The application of AgNPs at a concentration of 512 µg mL^−1^ resulted in a decrease in chlorophyll content by 11.8% (0.05 mg g FW^−1^) (**Figure 3**; **Supplementary Table S1**).

### 3.5 Oxidative stress parameters

Changes in the activity of individual components of the antioxidant system for the tested plants, concerning non-treated plantlets, given as percentages (%), are shown in **Supplementary Table S1**.

#### 3.5.1 Hydrogen peroxide content

**Figure 4:**
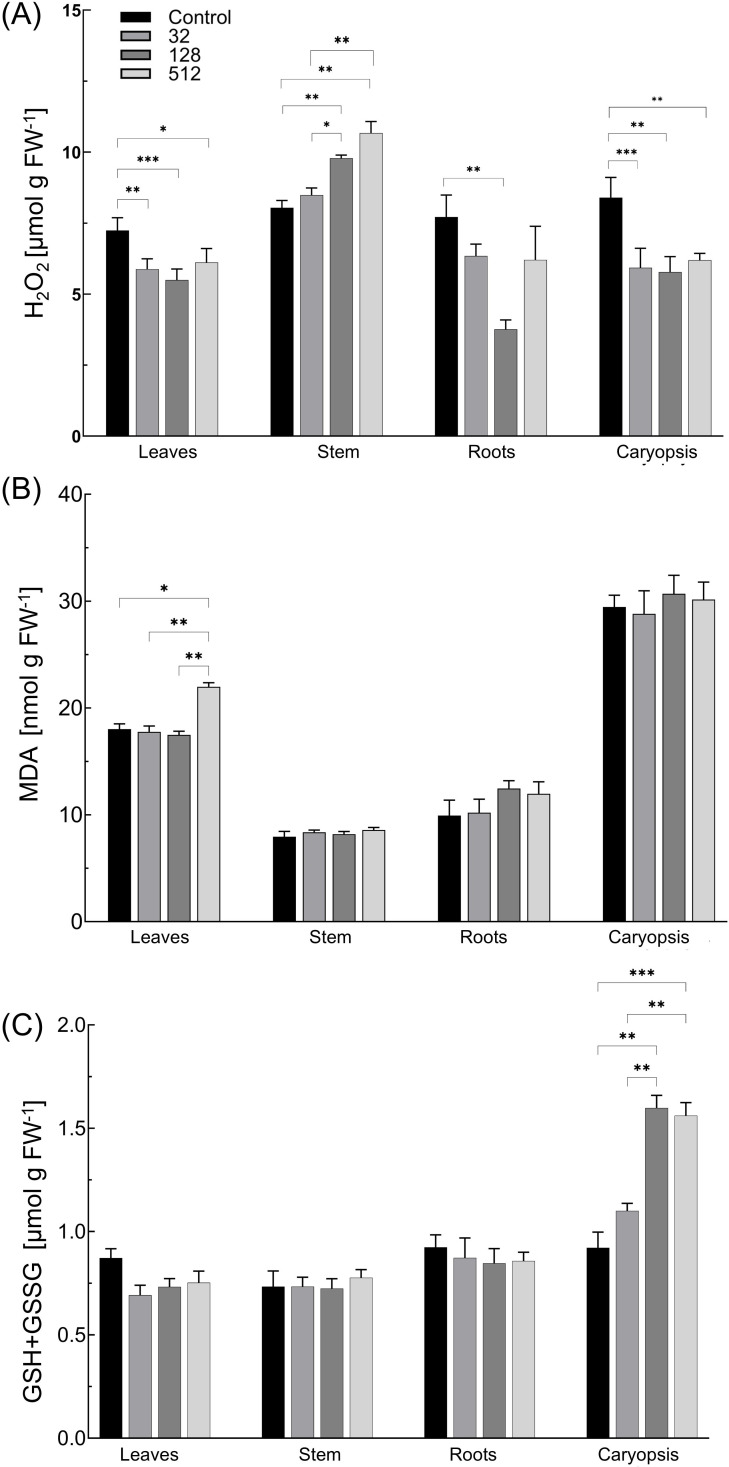
Influence of maize grain sterilization with AgNPs on levels of hydrogen peroxide (H_2_O_2_) **(A)**, malondialdehyde (MDA) **(B)**, and total glutathione (GSH+GSSG) **(C)** in 14-day-old maize plantlets (n=9). Data presented as mean and standard error (± SE) and statistical significance (*p*-value: *p ≤ 0.05, **p ≤ 0.01, and ***p ≤ 0.001).

The accumulation of H_2_O_2_ varied depending on the AgNPs concentration used and the plant organ (**Figure 4A**). In general, its level decreased after grain sterilization with AgNPs, and statistically lower concentrations of H_2_O_2_ were noted in caryopses by 26%–31% and in leaves by 16%–24%. In roots, the concentration of H_2_O_2_ was reduced by 17.7% (32 µg mL^−1^ AgNPs), 51.2% (128 µg mL^−1^ AgNPs), and 19.5% (512 µg mL^−1^ AgNPs). Simultaneously, in stems of maize plantlets treated with 128 and 512 µg mL^−1^ of AgNPs, the concentration of H_2_O_2_ increased by 21.7% and 32.7%, respectively.

#### 3.5.2 Lipid peroxidation

The level of MDA in all control plant organs and AgNPs-treated plantlets was comparable (**Figure 4B**). The MDA content increased slightly in roots (by 21%–26%) after seed treatment with 128 and 512 µg mL^−1^ of AgNPs, but the effect was not statistically significant. In contrast, a significant increase in the MDA level by 3.96 nmol g FW^−1^ (22%) was detected in the leaves of plants developed from grains treated with 512 µg mL^−1^ of AgNPs, when compared to control plants.

#### 3.5.3 Total glutathione content

The total glutathione content in leaves, stems, and roots was comparable between all tested variants, with no effect of the AgNPs treatment observed (**Figure 4C**). However, total glutathione levels gradually increased by 19.4%, 69.4%, and 73% in caryopses of plants treated with AgNPs at concentrations of 32, 128, and 512 µg mL^−1^, respectively.

#### 3.5.4 Reduced and total (reduced and oxidized) ascorbate contents and ascorbate redox ratio

**Figure 5:**
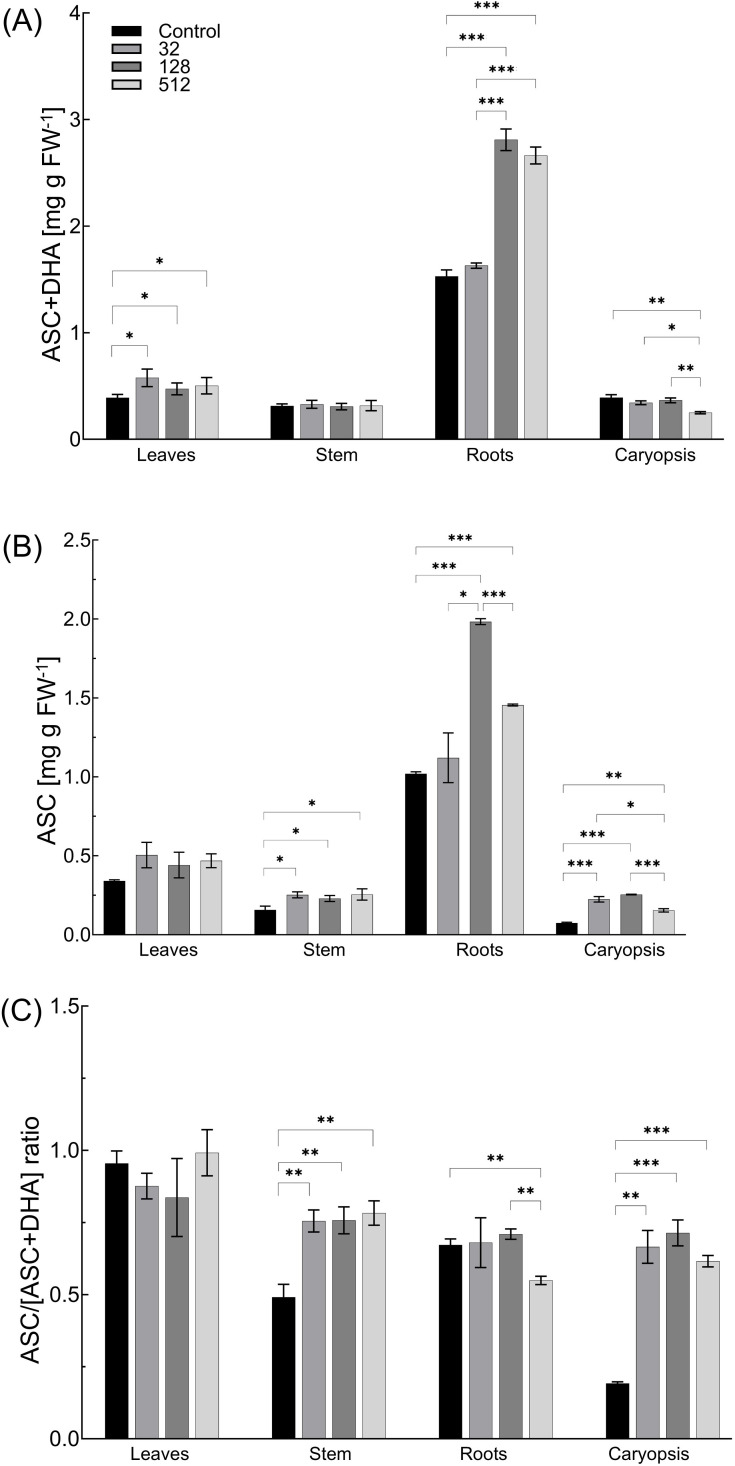
Influence of maize grain sterilization with AgNPs on the ascorbate plus dehydroascorbate (ASC+DHA) content **(A)**, ASC level **(B)**, and ascorbate redox state [ASC/(ASC +DHA) ratio] **(C)** of 14-day-old maize plantlets (n=9). Data presented as mean and standard error (± SE) and statistical significance (*p-*value: *p ≤ 0.05, **p ≤ 0.01, and ***p ≤ 0.001).

All AgNPs treatments increased leaf total ascorbate (ASC and DHA) content (by 21.5%–48.1%) and showed no significant impact on the ascorbate redox state (**Figure 5**). The increase in ASC content (by 45.5%–62.1%) and subsequently approximately 1.5-fold higher ASC/DHA ratios were observed in stems of plants treated with all AgNPs concentrations.

AgNPs at concentrations of 32 and 128 µg mL^−1^ showed a minor effect on the total ascorbate pool in caryopses, while at a concentration of 512 µg mL^−1^, the parameter was reduced by 36.3%. However, treating grains with AgNPs at concentrations of 32, 128, and 512 µg mL^−1^ increased the ascorbic acid concentration by 3, 3.4, and 2.1 times, respectively. Consequently, 3.5-, 3.8-, and 3.2-fold increases in ASC/DHA+ASC ratio in caryopses were observed under AgNPs treatment at concentration of 32, 128, and 512 μg mL^-1^, respectively.

Different concentration-dependent effects of AgNPs treatment on the overall ASC and DHA pool and redox status in roots were observed. Both total ascorbate and ascorbic acid contents were not altered after treatment with the lowest concentration of AgNPs (32 µg mL^−1^). However, the treatment of grains with AgNPs at a concentration of 128 µg mL^−1^ increased the total ascorbate pool and the content of its reduced form (ASC) by 83.8% and 94.4%, respectively. The ascorbate redox status was at the same level as in the control. In contrast, the treatment with the highest tested concentration of AgNPs (512 µg mL^−1^) increased the total ascorbate by 74.2% and the ASC content by 42.7%. The ascorbate redox status decreased, under the highest AgNPs concentration, by 18.3% when compared to the control (**Figure 5**).

#### 3.5.5 Activity of catalase

**Figure 6:**
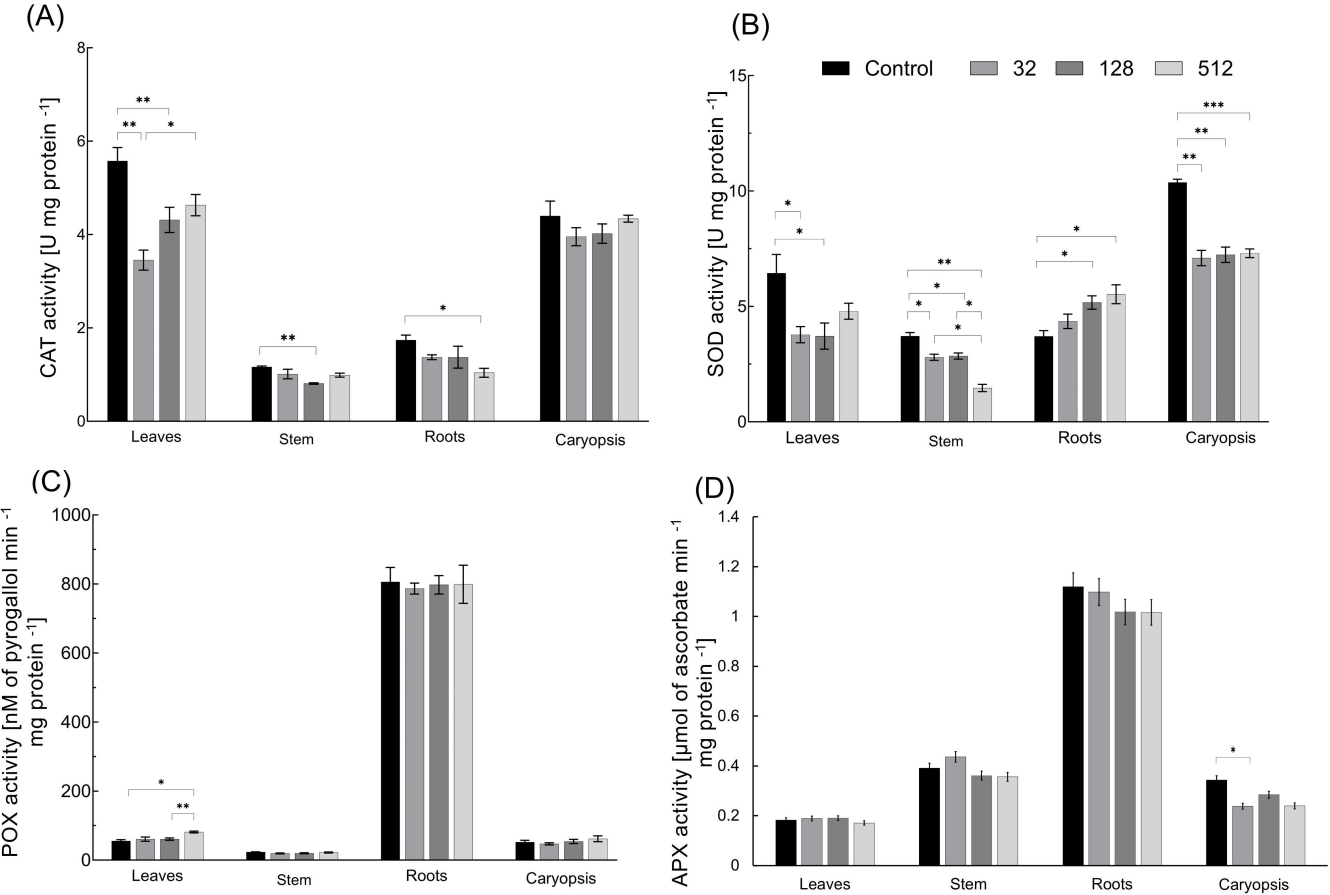
Influence of maize grain sterilization with AgNPs on the activity of catalase (CAT) **(A)**, superoxide dismutase (SOD) **(B)**, peroxidase (POX) **(C)**, and ascorbate peroxidase (APX) **(D)** in 14-day-old maize plantlets (n=9). Data presented as mean and standard error (± SE) and statistical significance (*p-*value: *p ≤ 0.05, **p ≤ 0.01, and ***p ≤ 0.001).

The treatment of grains with AgNPs resulted in the reduction of catalase (CAT) activity in all variants when compared to control plantlets (**Figure 6A**). A significantly lower CAT activity was revealed in the leaves of plants treated with 32 and 128 µg mL^−1^ of AgNPs, with enzyme activity decreasing by 38.1% and 22.6%, respectively. In stems of plantlets treated with 32 and 128 µg mL^−1^ of AgNPs, the activity of CAT was reduced by 38.1% and 22.6%, respectively. The reduction of CAT activity by 40.3% was observed in the roots of plants developed from grains treated with the highest tested concentration of AgNPs. The CAT activity in caryopses did not differ between the control and AgNPs-treated plants (**Figure 6A**).

#### 3.5.6 Activity of superoxide dismutase

SOD activity varied substantially between the control and AgNPs-treated variants, decreasing, in a AgNPs dose-dependent manner, in aboveground organs and caryopses and increasing in plant roots (**Figure 6B**). In leaves, SOD activity decreased by 41.2%, 42.4%, and 25.6% under treatments with 32, 128, and 512 µg mL^−1^ of AgNPs, respectively. In stems of plants, developed from grains treated with AgNPs at concentrations of 32, 128, and 512 µg mL^−1^, SOD activity decreased by 24.2%, 22.8%, and 60.3%, respectively. Reduced SOD activity by approximately 30%–32% was recorded in caryopses of plants treated with all tested concentrations of AgNPs. The SOD activity in roots increased by 16.6%–49.2%, with increasing AgNPs concentrations.

#### 3.5.7 Activity of peroxidases

The surface sterilization of maize grains with AgNPs had a minor impact on peroxidase (POX) activity in developed plantlet organs (**Figure 6C**). Significantly lower POX activity, by 46.6%, was observed in leaves after the use of AgNPs at a concentration of 512 µg mL^−1^. The activity of POX was slightly reduced in stems (by 16.8% and 14.9% after treatment with AgNPs at concentrations of 32 and 128 µg mL^−1^, respectively). The AgNPs at a concentration of 512 µg mL^−1^ increased POX activity in caryopses by 18.5%, but the differences did not pass the significance test.

#### 3.5.8 Activity of ascorbate peroxidase

APX activity was unaffected by AgNPs treatment in leaves, stems, and roots. The reduction in enzymatic activity of APX by 17% was found in caryopses after treatment with 128 and 512 µg mL^−1^ of AgNPs, whereas a 30.7% reduction occurred when grains were subjected to treatment with 32 µg mL^−1^ of AgNPs (**Figure 6D**).

### 3.6 Principal component analysis and hierarchical cluster analysis

**Figure 7:**
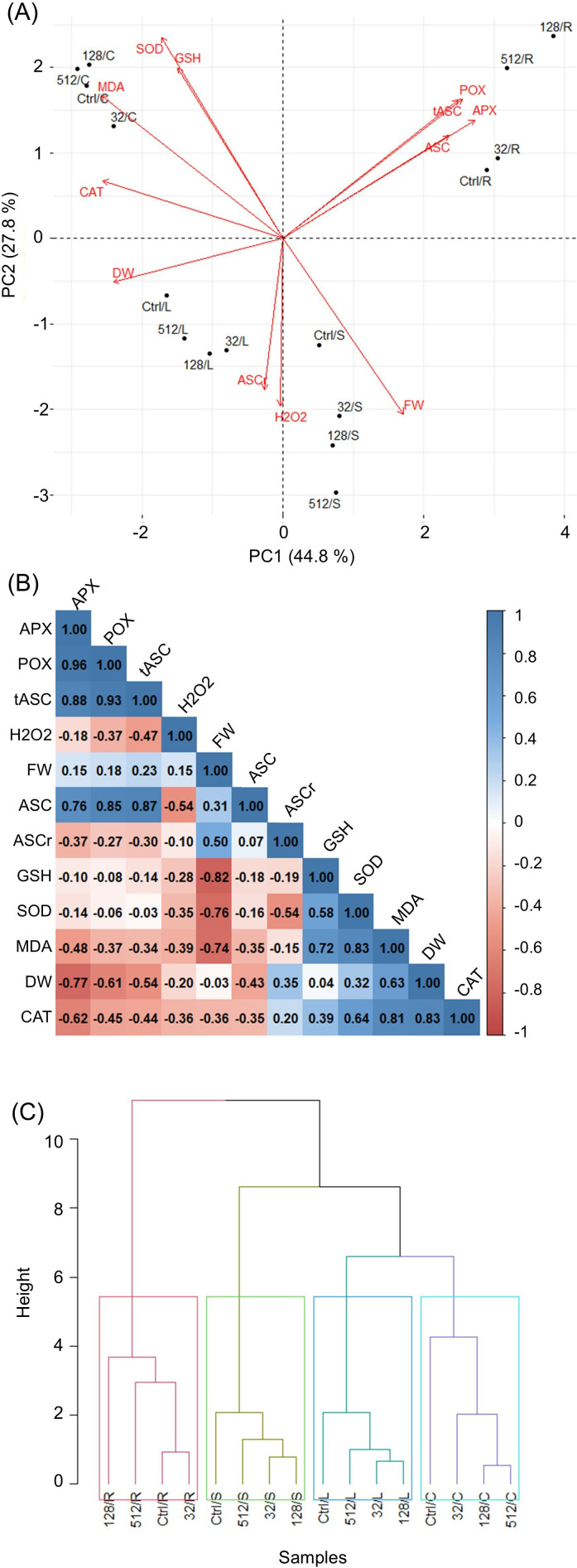
Analysis of general alterations and correlations of growth and individual biochemical parameters among organs of plantlets developed from grains treated with AgNPs. (PCA) Biplot **(A)**, where arrows indicate the strength of the trait influence on the first two PCs. Correlation analysis between all the studied parameters, where red and blue colors represent positive and negative correlations, respectively **(B)**. Dendrogram of hierarchical cluster analysis (HCA) showing associations in changes of biochemical parameters among various AgNPs treatments and maize plantlets organs **(C)**. APX, ascorbate peroxidase; ASC, ascorbate; ASCr, reduced ascorbate; DW, dry weight; FW, fresh weight; GSH, glutathione; H_2_O_2_, hydrogen peroxide; MDA, malondialdehyde; POX, peroxidase; SOD, superoxide dismutase; tASC, total ascorbate; Ctrl, untreated control; 32, treatment with AgNPs at concentration of 32 µg mL^−1^; 128, treatment with AgNPs at concentration of 128 µg mL^−1^; 512, treatment with AgNPs at concentration of 512 µg mL^−1^; L, leaves; S, stem; R, roots; C, caryopses; AgNPs, silver nanoparticles; PCA, principal component analysis.

The results of PCA and HCA showing general alterations and correlations of growth and individual biochemical parameters among organs of plantlets developed from grains treated with AgNPs are demonstrated in **Figure 7**. The first two components, PC1 and PC2, allow us to represent high values of the initial variability of the data (44.8% and 27.8%, respectively). The results showed that APX activity, MDA, and CAT activity showed the highest contribution to PC1 (14.0%, 12.6%, and 12.3%, respectively), while the contribution of variables to PC2 was explained by SOD activity (16.8%), FW (12.9%), and GSH content (12.2%) (**Supplementary Table S3**). The highest positive correlation was found for ASC and total ASC, along with POX and APX (**Figures 7A, B**). The effect of the individual concentrations of AgNPs (32, 128, and 512 µg mL^−1^) among plantlet organs (leaves, caryopses, stems, and roots) based on biochemical and growth parameters were assayed by HCA (**Figure 7C**). All organs were classified as separate groups, including untreated samples. In both leaves and stems, changes in biochemical and growth parameters under AgNPs treatments at concentrations of 32 and 128 µg mL^−1^ were nearest to each other, followed by concentrations of 512 µg mL^−1^ and controls. The caryopses showed dose-dependent alteration, where the two highest concentrations (128 and 512 µg mL^−1^) caused a similar response pattern, followed by a concentration of 32 µg mL^−1^ and controls. In roots, the effects of AgNPs treatment at the concentration of 32 µg mL^−1^ were found to be similar to those of controls, followed by concentrations of 128 and 512 µg mL^−1^.

## 4 Discussion

### 4.1 Biosynthesis and physicochemical characteristics of AgNPs from *F. solani* IOR 825

Biological synthesis of NPs addresses the need to develop environmentally friendly, efficient, and safe methods of producing NPs. Many studies have shown that fungi display potential for the biosynthesis of NPs, as they efficiently produce the biomass along with a variety of chemical compounds responsible for the reduction of silver ions to silver NPs and possess a high tolerance to metals (Borehalli Mayegowda et al., 2023). It was reported that fungal enzymes are involved in the synthesis of AgNPs (Ahmad et al., 2003; Hamedi et al., 2017). In a study conducted by El-Sayed and El-Sayed (2020), the protein-rich cell-free filtrate of *F. solani* KJ 623702 was successfully used for the synthesis of AgNPs, CuNPs, and ZnONPs.

The physicochemical properties of biosynthesized AgNPs and consequently their biological interactions or reactivity are highly dependent on synthesis conditions including the biological source of reducing and stabilizing agents, type, and concentration of precursor salt, as well as the temperature and pH of the reaction mixture (Sidhu et al., 2022; Trzcińska-Wencel et al., 2023a). The results of our study confirmed that the fungal strain *F. solani* IOR 825 is a system for the efficient synthesis of AgNPs with small size (10–50 nm) and the presence of natural origin capping biomolecules on their surface. This capping can be involved in the reduction of silver ions (Ag^+^) and the formation of AgNPs and affects their stability and antimicrobial activity. Moreover, the coating modulates the interaction between nanoparticles and biological surfaces, which affects the potential uptake of nanoparticles (Huang et al., 2022; Wypij et al., 2022). In accordance with our results, El-Sayed and El-Sayed (2020) found that FTIR analysis of AgNPs from *F. solani* KJ 623702 showed peaks attributed to N–H bending, C–H stretching vibrations of protein methylene groups, and O–H stretching of carboxylic acids. The authors, based on sodium dodecyl sulfate–polyacrylamide gel electrophoresis (SDS-PAGE) and FTIR results, suggested that the process of biogenic synthesis of NPs is related to the presence of various fungal-origin compounds (containing sulfur, nitrogen, and phosphorus), proteins with β-sheet and a carbonyl group of amino acid residues, and glycoprotein-containing polysaccharides with α-glycosidic bond (El-Sayed and El-Sayed, 2020). The nanoparticle surface properties such as surface charge and hydrophobicity/hydrophilicity are responsible for electrostatic repulsion between individual nanoparticles that prevent aggregation. This makes nanoparticles more mobile and more available for plants than bare nanoparticles. In contrast to chemically synthesized NPs, which are most frequently coated with polyvinylpyrrolidone (PVP), Arabic gum (AG), citrate, and cetyltrimethylammonium bromide (CTAB), the biological NPs are coated with molecules of natural origin (plants or microbial extracts). Consequently, the biological coating is considered less toxic than the chemical one (Sidhu et al., 2022; Wypij et al., 2022). Similar findings were described in our previous studies by Trzcińska-Wencel et al. (2023b, 2023c), who showed the potential of using the fungi of the genus *Fusarium* for the efficient, low-cost, simple, and environmentally friendly synthesis of metal nanoparticles with desirable physical and chemical properties to provide biocompatibility and biological activity as well as their potential for use in multiple applications, including agriculture.

### 4.2 Efficacy of AgNPs treatment on grain germination and growth parameters in maize

Although silver is not an essential element for plant growth as compared to other micronutrients such as copper, zinc, iron, or magnesium, its high antimicrobial activity is an advantage in many applications (Duffy et al., 2018; Kakian et al., 2024). The nanoscale form of silver can provide their slow and controlled release over time, thus prolonging antimicrobial protection for plants (Bernardo-Mazariegos et al., 2019; Cao et al., 2025). Moreover, as the biogenic AgNPs exhibit higher antimicrobial activity than biogenic CuNPs, ZnNPs, and FeNPs, their use at a lower effective dose can reduce the toxicity of nanoproducts released into the environment (Asghar et al., 2018; Trzcińska-Wencel et al., 2023b). Therefore, biosynthesized AgNPs are excellent alternatives to conventional fungicides, which are based on copper compounds (Ding et al., 2019; Yen et al., 2019; Khan et al., 2021; Trzcińska-Wencel et al., 2024).

The germination of seeds and early growth of seedlings are identified as critical stages in the development and establishment of plants (Rajjou et al., 2012). A high threat of microbial contamination of seeds used for sowing provides a risk of fungal pathogen growth and infection of germinating seeds or developing seedlings (Magan et al., 2004). Seed priming is an effective method to prepare seeds for sowing to increase germination efficiency and achieve improved seedling growth (Abbasi Khalaki et al., 2021). As previously reported by Trzcińska-Wencel et al. (2023b), the use of AgNPs for grain pretreatment eliminated grain-borne pathogens at the lowest effective AgNPs concentration of 32 µg mL^−1^ with no impact on grain germination efficiency. In the present paper, we decided to broaden the study on the effect of AgNPs on grain germination and plantlet growth using the effective concentration (32 µg mL^−1^), its fourfold higher concentrations (128 and 512 µg mL^−1^), and prolonged (14 days) plant growth period. Simultaneously, the disinfecting effect of the used AgNPs concentrations on grains was confirmed, as no microbial growth was visually detected on the surface of the germinating grains or plant growth medium (**Supplementary Figure S2**). The results showed no impact of all tested AgNPs concentrations on grain germination when compared with control. Nonetheless, the treatment accelerated plantlet development, as manifested by the improved growth of shoot and reduction of grain weight in comparison with the control, indicating a stimulatory effect of the AgNPs on maize development. Similarly, seed pre-sowing treatments with bio-AgNPs improved the growth of licorice (*Glycyrrhiza glabra*) (Kim et al., 2023), maize (*Z. mays*) (Karim et al., 2023), onion (*Allium cepa* L.) (Acharya et al., 2019), watermelons (*Citrullus lanatus*) (Acharya et al., 2020), winged bean (*Psophocarpus tetragonolobus*) (Kamal Kumar et al., 2020), wheat (*Triticum aestivum*) (Mondéjar-López et al., 2023), and black gram [*Vigna mungo* (L.) Hepper] (Krishnasamy et al., 2024). In the study reported by Soliman et al. (2020), seed priming with increasing AgNPs concentrations gradually improved the germination of seeds and growth of maize (*Z. mays* L.), fenugreek (*Trigonella foenum-graecum* L.), and onion (*A. cepa* L.). Moreover, stimulatory effects on the growth and development of seedlings after seed nanopriming with the AgNPs synthesized using chemical and physical methods were reported for common beans (*Phaseolus vulgaris*) (Savassa et al., 2021) and beans (Prażak et al., 2020). However, in the other experimental system, namely, medium supplementation with chemically synthesized nanoparticles at a concentration of 100 ppm, the phytotoxic effects were observed on *Bacopa monnieri* (Krishnaraj et al., 2012). Higher phytotoxicity was observed under foliar application of AgNPs than root exposure in soybean and rice (Li et al., 2017), while the application of PVP-coated AgNPs resulted in limited germination and a decrease in the growth of *T. aestivum* L. (Vannini et al., 2014).

As numerous studies have suggested the adverse effects of NPs (especially at high concentrations), there is a need for extensive research on the interactions that occur between NPs and plants (Ding et al., 2019; Khan et al., 2021; Trzcińska-Wencel et al., 2024). The bioaccumulation and translocation of AgNPs in the treated plants depend on the plant (e.g., species and stage of growth), method of application, time of exposure, or dose used. The biological activity of AgNPs is determined by their physicochemical features, mainly by size, chemical composition, and surface properties. These parameters influence the uptake and transport of NPs within plant organs, their interactions with cellular components (e.g., cell wall penetration), and the release of silver ions, subsequently contributing to their effect on plant growth (Nandini et al., 2023; Trzcińska-Wencel et al., 2024). AgNPs in the environment undergo various biotransformations such as aggregation, ion release, sulfidation, or complexation with organic matter that determine their bioavailability to plants or toxicity. It has been noticed that AgNPs transport throughout the plant tissues via both apoplastic and symplastic pathways (Ali et al., 2021; Cao et al., 2025). However, our study showed that the Ag was undetected in any of the plantlet organs after 14 days of pre-sowing treatment of grains. In contrast, in a study conducted by Savassa et al. (2021), the seed treatments with 1, 10, and 100 mg L^−1^ of chemically synthesized AgNPs, Ag_2_SNPs, and AgNO_3_ (control) resulted in silver accumulation and biotransformation in *P. vulgaris* seeds. Ag was detected in the seed coat depending on the Ag source and concentration used. After 5 days from seed treatment, the Ag from AgNO_3_ was detected in the epidermis, while the Ag from AgNPs and Ag_2_SNPs was mainly located in the internal layer (parenchyma cells) of seeds. The study of Koley et al. (2023) implies that seed treatment with AgNPs led to their accumulation at a low dose and then the gradual release of Ag^+^ ions, which stimulated the antioxidant system with accelerated germination and growth of legume plants. In turn, Yan et al. (2023) reported that AgNPs stimulated the germination process of rice seeds due to the AgNPs uptake during the imbibition process. AgNPs were accumulated in decreasing concentrations in the seed coat, embryo, and endosperm. In contrast, the metabolomic and transcriptomic analyses revealed that AgNPs increased in stress signaling molecule synthesis, showing their potential as nanobiostimulators to ensure long-term stress memory (Yan et al., 2023).

### 4.3 Effect of AgNPs treatment on oxidative stress parameters and antioxidant system response

AgNPs may induce ROS generation and strengthen antioxidant system response by the enzymatic and/or non-enzymatic pathways involving both a wide range of enzymes (e.g., superoxide dismutase, catalase, peroxidases) and low-molecular-weight compounds (e.g., ascorbic acid, glutathione, proline, and tocopherols) (Sharma et al., 2019). In the present study, the treatment of maize grains with AgNPs specifically affected cellular redox agents in plant organs subjected to analysis. Symptomatically, as revealed by the lipid peroxidation assay, the treatments did not cause severe oxidative stress in any organ. This finding shows that optimal AgNPs treatments do not increase the risk of oxidative damage. However, the slight but significant increase in the rate of MDA formation and a decrease in total chlorophyll content occurred in leaves, but solely in plants challenged with the highest AgNPs concentration. Similarly, the decrease in photosynthetic pigments was reported in *Brassica* sp. seedlings exposed to AgNPs synthesized from *Aloe vera* extract (Vishwakarma et al., 2017) and in *Lupinus termis* after exposure to AgNPs synthesized from the leaf extract of *Coriandrum sativum* (Al-Huqail et al., 2018). In our study, the photosynthetic rate and the maximum efficiency of photosystem II were not analyzed in AgNPs-treated maize plants. Therefore, it remains to be determined to what extent the decrease in chlorophyll content affects photosynthetic efficiency. However, this issue will be addressed in our forthcoming study. Other authors have shown that a decrease in chlorophyll content adversely affects photosynthesis. Under decreased chlorophyll levels, excess electron flow may result in an imbalance between the donor and acceptor sites of photosystem II. This results in molecular oxygen reduction generating harmful ROS and photosystem damage (photoinhibition) (Bhattacharjee, 2019). The increase in lipid peroxidation and decrease in chlorophyll content in leaves of plants exposed to the highest AgNPs concentration may be related to increased photoinhibition occurring at excessive nanoparticle concentration. Although further experiments are required to assess if maize leaves become more susceptible to photooxidation under non-optimal AgNPs concentrations, these findings stress the necessity of optimizing the treatment procedure thoroughly since treating seeds with excessive AgNPs concentrations may bring about some detrimental consequences.

Although no significant oxidative damage was detected in AgNPs-treated plants, several components of the cellular redox systems were affected by treatments. Usually, the parameters were altered in an organ-specific manner. In leaves, the H_2_O_2_ levels were moderately reduced in the AgNPs-treated plants. The decrease in the oxidant content may be partly due to a substantial reduction in the activity of SOD, which is a H_2_O_2_-producing enzyme. The stable total glutathione levels and a small increase in the ASC content may also contribute to reducing the H_2_O_2_ level in leaves. Therefore, the ascorbate is supposed to scavenge H_2_O_2_ in a non-enzymatic manner since no increase in the APX activity can be observed (Kunert and Foyer, 2023). Gupta et al. (2018) evaluated the stimulatory effects of AgNPs on rice seedlings and suggested that growth promotion was related to efficient ROS scavenging mechanisms, including changes in glutathione–ascorbate cycle and activities of involved enzymes (ascorbate peroxidase and glutathione reductase) in leaves. Contrary to our results, authors have suggested low alterations of SOD activity in response to AgNPs treatment. Soliman et al. (2020) reported an increase in ascorbate and glutathione contents in leaves of *Z. mays*, *A. cepa*, and *T. foenum-graecum*, as well as an increase in expression levels and activities of antioxidant enzymes in AgNPs-treated seedlings. In turn, our results indicate that the H_2_O_2_-scavenging enzymes, namely, POX and CAT, differentially reacted to AgNPs treatment. A negligible increase in POX activity and a substantial decrease in CAT activity were observed. When compared to APX and other H_2_O_2_-scavenging enzymes, CAT has a low affinity to its substrate. Therefore, the enzyme is involved in H_2_O_2_ scavenging when oxidants are accumulated to high levels (Heck et al., 2010; Černý et al., 2018). Since the H_2_O_2_ content decreased under the AgNPs treatment, the amount of the enzyme may be adjusted accordingly by decreasing its abundance. Earlier studies demonstrated that CAT activity is dependent on the high availability of H_2_O_2_ (Scandalios et al., 1983; Rodríguez-Ruiz et al., 2019).

The growth of the AgNPs-treated plant roots was maintained at the control level. The H_2_O_2_ content was decreased in roots. This may be linked to the rise in the level of the H_2_O_2_-scavenging agent, namely, ascorbate. Furthermore, increased ascorbate content was accompanied by high activities of the H_2_O_2_-consuming enzymes, namely, POX and APX. At this stage of plant development, significant root growth is observed that is correlated with a wide range of physiological processes, such as auxin metabolism, cross-linking of cell wall components, or cell elongation, in which peroxidases are involved (Passardi et al., 2005; Majda and Robert, 2018). POX is a prevalent apoplast-targeted enzyme. However, the majority of ascorbate is located in the cytosol, where APX isoforms are present. Therefore, we believe that the increasing ASC levels in the roots of the AgNPs-treated plants may contribute to maintaining high APX activity, which in turn prevents excessive H_2_O_2_ accumulation. These mechanisms may be responsible for the reduction in H_2_O_2_ content in the roots of the AgNPs-treated plants. In contrast, decreased CAT activity and increased SOD activity may favor H_2_O_2_ accumulation (Kumari et al., 2021). Acting simultaneously, the aforementioned factors may contribute to H_2_O_2_ homeostasis in roots under the AgNPs treatment. In turn, Kim et al. (2023) observed improvements in shoot and root elongation of mung bean (*Vigna radiata*) after 12-hour seed treatment with biosynthesized AgNPs (at concentrations of 12.5, 25, and 50 ppm). In contrast to our findings, other authors suggested inhibition in root development after direct exposure of roots to AgNPs in soil or medium (Cvjetko et al., 2017). In the study reported by Guilger-Casagrande et al. (2022), the effect of soil exposure of soybean seedlings to AgNPs resulted in a reduction in dry weight. In addition, the increase in H_2_O_2_ and lipid peroxidation (higher MDA content) in seedling roots was observed. Considering the inconclusive results, further research is still required to understand the effects of AgNPs on root development in plants in view of different species and the conditions of NPs application.

The stimulatory effect of AgNPs treatments on maize was observed as the reduction in biomass of the caryopses. This was accompanied by a consistent decrease in H_2_O_2_ content and APX, CAT, and SOD activities. Simultaneously, the redox balance of ascorbate turned out to be further reduced, and the content of total glutathione substantially increased. The latter effect was more pronounced under treatments with higher AgNPs concentrations. The increase in total glutathione concentration significantly distinguished the caryopses from other organs. Glutathione is a versatile molecule, with many functions that go beyond common antioxidant roles. Glutathione is a source of sulfur for protein biosynthesis, plays a crucial role as an antioxidant, and represents the potential as a highly reducing chemical barrier to prevent over-oxidation of cellular components through its direct interaction with peroxides or as a substrate for ROS-neutralizing enzymes (Noctor et al., 2012). Since ASC levels were relatively low in caryopses, GSH is supposed to be a major low-molecular-weight antioxidant in this organ. Consequently, the reduction in H_2_O_2_ level in caryopses may be a consequence of AgNPs-dependent accumulation of GSH in this organ. It is suggested that the detoxification of Ag in plant cells is related to the direct bonding of Ag with GSH through the −SH group or utilization of GSH for increased production of phytochelatins involved in metal detoxification (Kaur et al., 2021; Larue et al., 2014). Enhanced antioxidant protection in the caryopses, due to AgNPs-stimulated GSH accumulation, may be important for mitigating the relatively high oxidative damage in this organ, as exemplified by high lipid peroxidation. However, the latter is not related to AgNPs treatment. It should be kept in mind that at this developmental stage, plant growth is still important and supported by seed reserves (Kennedy et al., 2004). Therefore, a complete analysis of the metabolic status of caryopses in a 2-week-old maize plant is required to interpret the behavior of redox agents analyzed in this study.

Our study shows that the activities of certain enzymes decreased at AgNPs treatment. Studies on plants overexpressing CAT, APX, or SOD have shown that these enzymes act synergistically to keep the homeostasis of cellular redox state, being increased or decreased in an orchestrated manner (Scandalios et al., 1983; Faize et al., 2011; Wang et al., 2017; Che et al., 2020). We suspect that the decrease in the enzyme activities, observed in all organs except the stem, may reflect their synchronized response to a decrease in H_2_O_2_ resulting from the AgNPs treatment. In stems, the increase in the H_2_O_2_ content was in line with the enhanced stem biomass gain and stem lengthening resulting from AgNPs administration. Therefore, we assume that it may be a growth-related process, involving rather an apoplast H_2_O_2_, whereas CAT is a cytosolic enzyme and may not directly respond to changing apoplast H_2_O_2_ levels (Majda and Robert, 2018; Marzol et al., 2022). In this organ, H_2_O_2_ homeostasis may be regulated by the ascorbate pool, which turned out to be more reduced when challenged with AgNPs.

**Figure 8:**
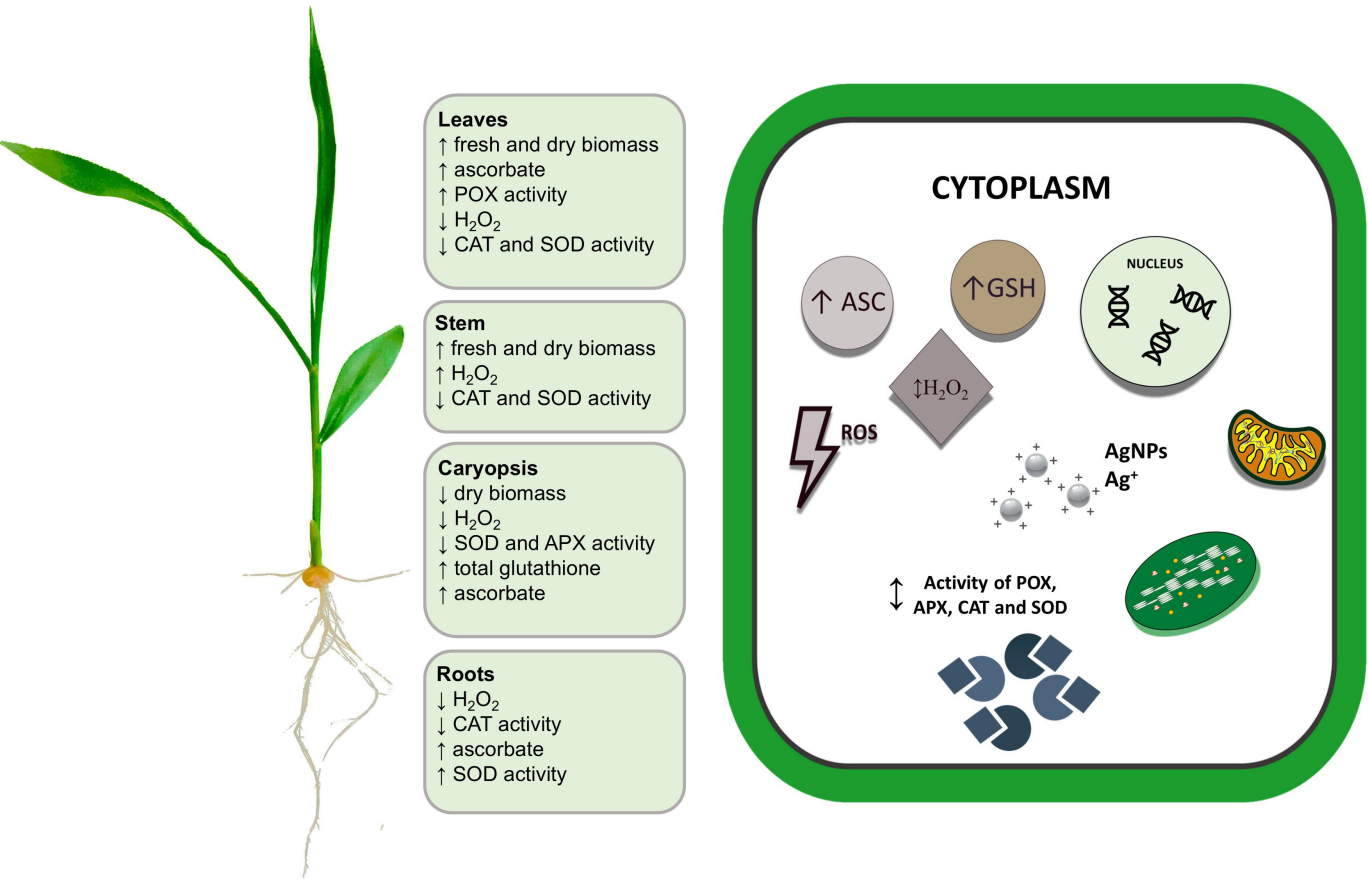
The summarized effects of AgNPs on maize growth and redox metabolism in maize plantlet organs. Upright- and downward-pointing arrows denote stimulatory and inhibitory effects of AgNPs treatments, respectively (for details, see text). APX, ascorbate peroxidase; ASC, ascorbate; CAT, catalase; GSH, total glutathione; H_2_O_2_, hydrogen peroxide; POX, peroxidase; SOD, superoxide dismutase.

To sum up, the possible mechanisms of AgNPs action in plantlets that are responsible for alterations in cellular redox metabolism during the AgNPs-dependent maize growth stimulation are presented in **Figure 8**. The overall decrease in H_2_O_2_ accumulation in all organs (except stem) suggests that the efficiency of redox reactions has increased after grain treatments with AgNPs. The results indicate that specific plantlet organs showed a varying response and that the induced effect depended on the applied dose. The alterations of the determined oxidative stress parameters were stronger with the application of higher AgNPs concentrations to grains. However, our results show no evidence of the induction of severe oxidative stress by AgNPs in maize plantlets, as overproduction of ROS and its consequences due to the incapability of an antioxidative defense system for efficient ROS scavenging were not detected.

## 5 Conclusions

To summarize, AgNPs synthesized from *F. solani* IOR 825 were applied for the pretreatment of maize grains to sterilize their surface and improve plant growth and development. Biogenic AgNPs showed potential for application in seed priming, which is linked to their remarkable antimicrobial activity even at low concentrations against grain-borne microbial pathogens that cause infections during germination and seedling development. The positive effect of AgNPs on shoot elongation and enhanced biomass of maize plantlets, without any negative impact on oxidative stress or the chlorophyll content, confirmed their crop-safe biostimulatory potential. Furthermore, the biosynthesis process and pre-sowing grain technique are simple, cost-effective, and environmentally friendly, indicating that they are affordable and implementable for practical use.

## Data Availability

The original contributions presented in the study are included in the article/**Supplementary Material**. Further inquiries can be directed to the corresponding authors.
