# Supplementary material for: Pre-sowing grain treatment with bio-AgNPs stimulates plant growth and affects redox homeostasis in maize
Source: Front Plant Sci. 2025 May 22;16:1494741. doi: 10.3389/fpls.2025.1494741 (PMC12137326; doi:10.3389/fpls.2025.1494741)
Supplement: Supplementary file 1 [file DataSheet1.docx]

Supplementary Material

**Pre-sowing grain treatment with bio-AgNPs stimulates plant growth and affects redox homeostasis in maize**

**Trzcińska-Wencel Joanna^1*^, Mucha Natalia^2^, Rai Mahendra^3,4^ Tyburski Jarosław^2^, Golińska Patrycja^1*^**

^1^ Department of Microbiology, Faculty of Biological and Veterinary Sciences, Nicolaus Copernicus University in Toruń, Toruń, Poland,

^2^ Department of Plant Physiology and Biotechnology, Faculty of Biological and Veterinary Sciences, Nicolaus Copernicus University in Toruń, Toruń, Poland

^3^ Nanobiotechnology Laboratory, Department of Biotechnology, SGB Amravati University, Amravati, India

^4^Department of Chemistry, Federal University of Piaui (UFPI), University Campus Ministro Petrônio Portella, Ininga, Teresina, Piauí, 64049-550, Brazil

*** Correspondence:**Trzcińska-Wencel Joanna
[trzcinska@doktorant.umk.pl](mailto:trzcinska@doktorant.umk.pl)

Golińska Patrycja
[golinska@umk.pl](mailto:golinska@umk.pl)

**
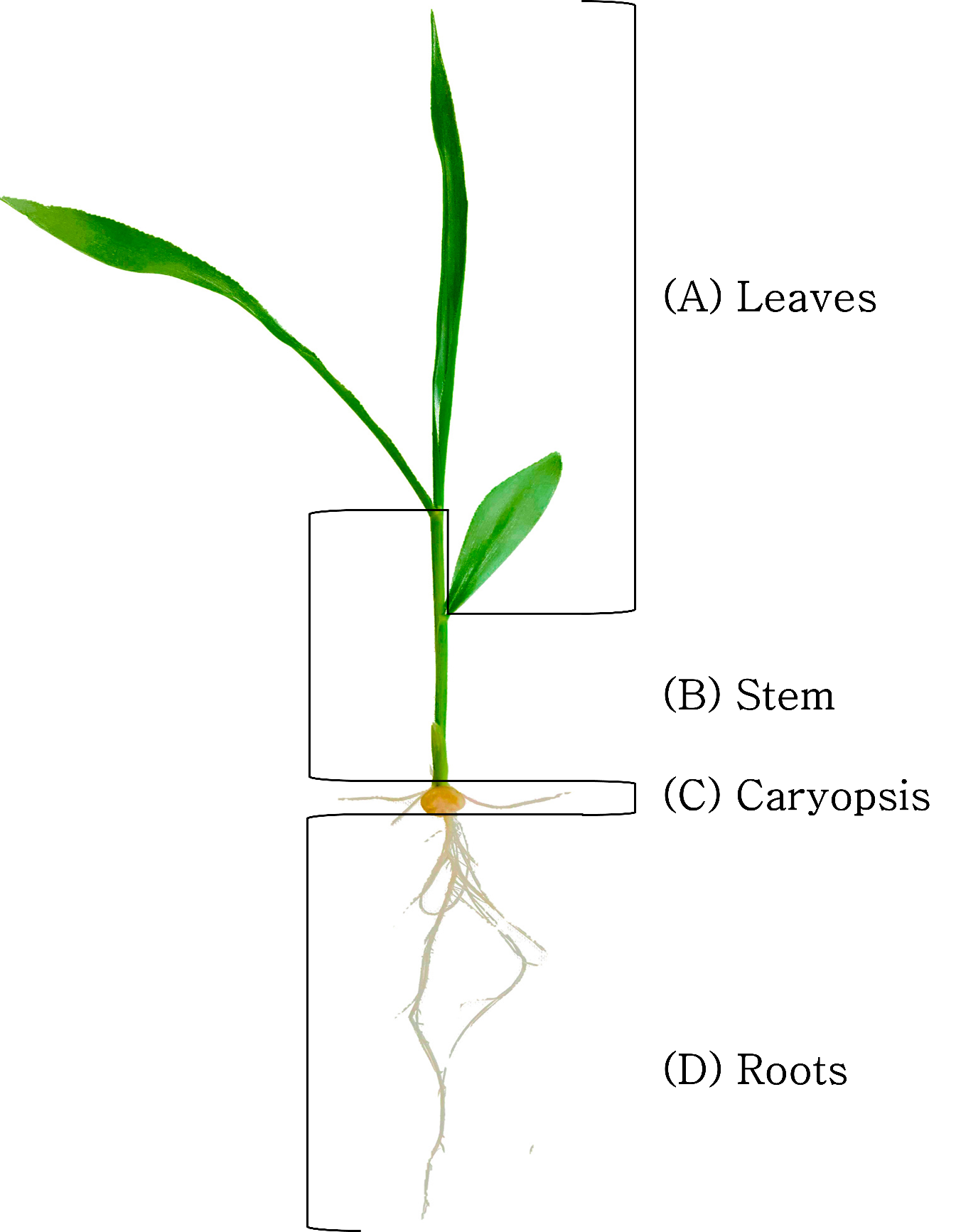
**

**Supplementary Figure S1.** Schematic diagram of a 14-day-old maize showing the individual organs (leaves, stem, caryopsis and roots) used to assess the activity of the antioxidant system.


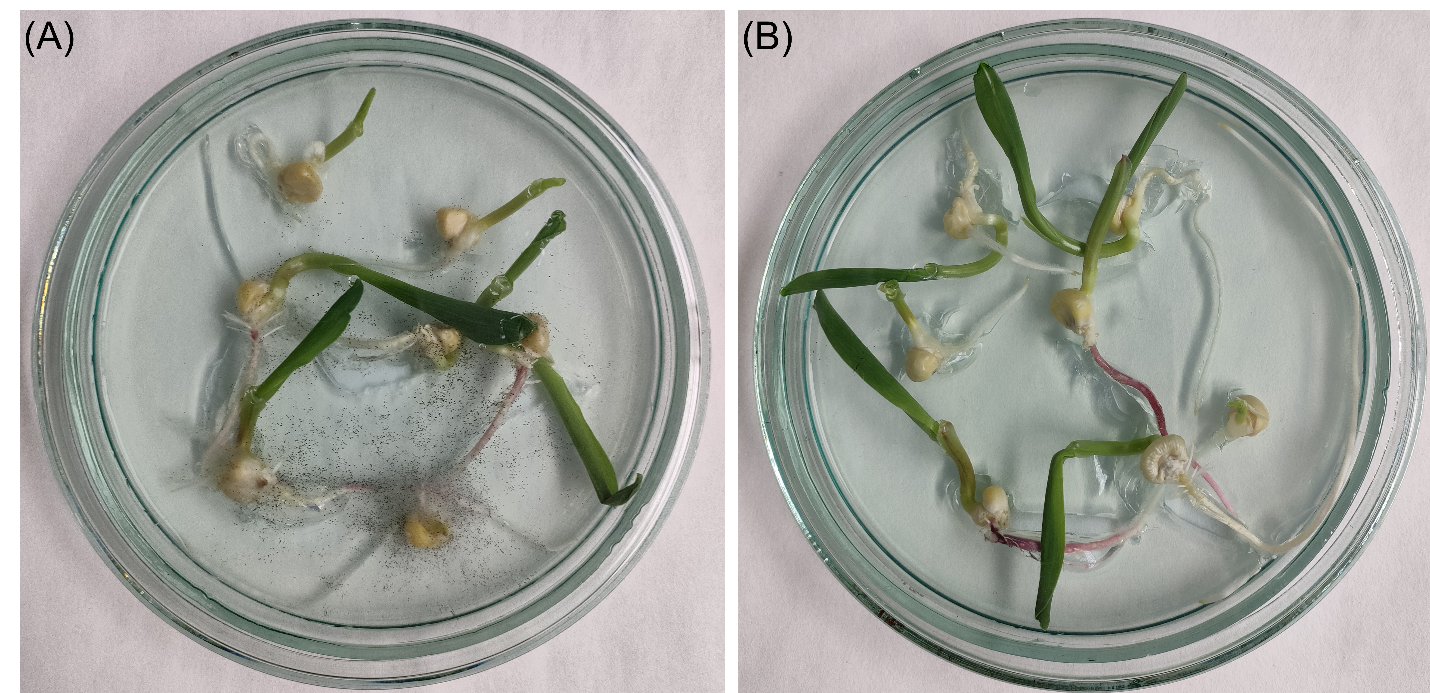


**Supplementary Figure S2.** The sterilization effect of AgNPs from *Fusarium solani* IOR825 on maize grains. The germinated unsterilized grains treated with sterile distilled water (without AgNPs) with visible microbial growth (A) and grains treated with AgNPs solution at the concentration of 32 µg mL^-1^ (B).

**Supplementary Table 1.** The summarized alterations [%] in growth parameters, accumulation of H_2_O_2_, malondialdehyde (MDA), total glutathione, total ascorbate and reduced ascorbate and enzymatic activity in individual organs of maize plantlets treated with bio-AgNPs in comparison with the control.

|  |  | |  |
| --- | --- | --- | --- |
|  | **AgNPs concentration [µg mL^-1^]** | | |
|  | **32** | **128** | **512** |
| **Lenght** |  |  |  |
| Shoot | 7.9 | 7.5 | 13.3* |
| Root | 9.2 | 5.8 | 11.0 |
| **Fresh weight** |  |  |  |
| leaves | 8.8 | 13.3 | 22.3* |
| stem | 25.5 | 27.1 | 39.2* |
| roots | 15.9 | 11.8 | 15.6 |
| caryopsis | -20.0 | -14.9 | -20.8* |
| **Dry weight** |  |  |  |
| leaves | 29.5* | 28.9* | 37.8* |
| stem | 12.4 | 24.2 | 43.1* |
| roots | 15.2 | 12.4 | 10.1 |
| caryopsis | -20.9* | -22.9* | -20.9* |
| **Chlorophyll content** |  |  |  |
| Leaves | 0.4 | -4.9 | -11.8* |
| **H_2_O_2_ concentration** |  |  |  |
| leaves | -18.8* | -24.1* | -15.5* |
| stem | 5.5 | 21.7* | 32.7* |
| roots | -17.7 | -51.2* | -19.5 |
| caryopsis | -29.4* | -31.2* | -26.2* |
| **MDA concentration** |  |  |  |
| leaves | -1.4 | -3.0 | 22.0* |
| stem | 5.3 | 3.0 | 7.9 |
| roots | 2.7 | 25.5 | 20.5 |
| caryopsis | -2.2 | 4.2 | 2.4 |
| **Total glutathione** |  |  |  |
| leaves | -20.7 | -16.0 | -13.8 |
| stem | 0.0 | -1.3 | 5.9 |
| roots | -5.5 | -8.4 | -7.1 |
| caryopsis | 19.4 | 73.5* | 69.4* |
| **Total ascorbate** |  |  |  |
| leaves | 48.1* | 21.5* | 29.1* |
| stem | 4.7 | -2.0 | 0.8 |
| roots | 6.6 | 83.8* | 74.2* |
| caryopsis | -12.5 | -6.7 | -36.3* |
| **Reduced ASC** |  |  |  |
| leaves | 47.9* | 29.3* | 37.3* |
| stem | 60.5 | 45.9 | 62.1 |
| roots | 9.9 | 94.4* | 42.7* |
| caryopsis | 201.2* | 241.7* | 107.3* |
| **CAT activity** |  |  |  |
| leaves | -38.1* | -22.6* | -17.0 |
| stem | -13.0 | -30.5* | -15.0 |
| roots | -21.1 | -21.1 | -40.3* |
| caryopsis | -10.1 | -8.4 | -1.3 |
| **SOD activity** |  |  |  |
| leaves | -41.4* | -42.4* | -25.6 |
| stem | -24.2* | -22.8* | -60.3* |
| roots | 17.6 | 39.6* | 49.2* |
| caryopsis | -31.6* | -30.2* | -29.6* |
| **POX activity** |  |  |  |
| leaves | 9.2 | 9.2 | 46.4* |
| stem | -16.8 | -14.9 | -5.4 |
| roots | -2.4 | -1.1 | -0.9 |
| caryopsis | -9.6 | 3.3 | 18.5 |
| **APX activity** |  |  |  |
| leaves | 3.3 | 4.4 | -6.6 |
| stem | 11.4 | -7.9 | -9.1 |
| roots | -1.9 | -9.0 | -2.7 |
| caryopsis | -30.7* | -17.2 | -17.8 |

**Supplementary Table S2.** The content of elements in the organs of 14-day-old maize treated with AgNPs determined by EDS analysis, data presented in mass %.

| AgNPs concentration [µg mL^-1^] / organ | | C | N | O | Na | Mg | Al | Si | P | S | Cl | K | Ag |
| --- | --- | --- | --- | --- | --- | --- | --- | --- | --- | --- | --- | --- | --- |
| 512 L | Mean | 12.1 | 5.57 | 73.53 | 0.00 | 0.19 | 0.69 | 0.03 | 1.02 | 0.39 | 1.06 | 5.28 | 0.13 |
|  | SD | 1.89 | 0.8 | 1.34 | 0.00 | 0.09 | 0.26 | 0.05 | 0.51 | 0.2 | 0.46 | 1.65 | 0.1 |
|  | SE | 0.95 | 0.4 | 0.67 | 0.00 | 0.04 | 0.13 | 0.03 | 0.25 | 0.1 | 0.23 | 0.83 | 0.05 |
| 512 S | Mean | 9.37 | 6.66 | 72.96 | 0.19 | 0.47 | 0.34 | 0.000 | 0.76 | 0.28 | 1.45 | 6.78 | 0.09 |
|  | SD | 1.65 | 1.14 | 1.91 | 0.44 | 0.33 | 0.32 | 0.010 | 0.24 | 0.15 | 0.4 | 1.16 | 0.1 |
|  | SE | 0.74 | 0.51 | 0.85 | 0.19 | 0.15 | 0.14 | 0.000 | 0.11 | 0.07 | 0.18 | 0.52 | 0.04 |
| 512 R | Mean | 6.87 | 6.61 | 68.74 | 1.51 | 0.5 | 0.55 | 0.21 | 0.83 | 1.81 | 1.1 | 10 | 0.27 |
|  | SD | 1.96 | 0.67 | 3.14 | 0.62 | 0.33 | 0.22 | 0.17 | 0.37 | 0.88 | 0.36 | 5.7 | 0.15 |
|  | SE | 0.74 | 0.25 | 1.19 | 0.23 | 0.12 | 0.08 | 0.07 | 0.14 | 0.33 | 0.14 | 2.16 | 0.06 |
| 512 C | Mean | 14.78 | 4.8 | 77.97 | 0.63 | 0.3 | 0.41 | 0.00 | 0.17 | 0.14 | 0.16 | 0.6 | 0.06 |
|  | SD | 0.49 | 0.17 | 0.36 | 0.04 | 0.05 | 0.08 | 0.00 | 0.05 | 0.04 | 0.05 | 0.07 | 0.03 |
|  | SE | 0.24 | 0.09 | 0.18 | 0.02 | 0.03 | 0.04 | 0.00 | 0.02 | 0.02 | 0.03 | 0.04 | 0.01 |
| 32 C | Mean | 15.62 | 3.37 | 76.99 | 0.53 | 0.41 | 0.62 | 0.07 | 0.43 | 0.18 | 0.28 | 1.08 | 0.07 |
|  | SD | 2.54 | 2.41 | 2.53 | 0.62 | 0.46 | 0.33 | 0.15 | 0.38 | 0.18 | 0.28 | 0.55 | 0.09 |
|  | SE | 1.27 | 1.21 | 1.26 | 0.31 | 0.23 | 0.17 | 0.07 | 0.19 | 0.09 | 0.14 | 0.27 | 0.04 |
| 128 C | Mean | 14.8 | 3.63 | 77.6 | 0.79 | 0.35 | 0.89 | 0.04 | 0.24 | 0.25 | 0.25 | 1.02 | 0.06 |
|  | SD | 1.6 | 1.51 | 1.2 | 0.36 | 0.22 | 0.66 | 0.07 | 0.11 | 0.12 | 0.06 | 0.12 | 0.05 |
|  | SE | 0.8 | 0.75 | 0.6 | 0.18 | 0.11 | 0.33 | 0.04 | 0.06 | 0.06 | 0.03 | 0.06 | 0.03 |
| 0  C | Mean | 17.8 | 2.95 | 75.2 | 0.5 | 0.29 | 1.00 | 0.6 | 0.22 | 0.17 | 0.16 | 0.69 | 0.03 |
|  | SD | 2.68 | 2.17 | 2.63 | 0.28 | 0.15 | 1.25 | 1.17 | 0.08 | 0.05 | 0.06 | 0.13 | 0.04 |
|  | SE | 1.2 | 0.97 | 1.18 | 0.12 | 0.07 | 0.56 | 0.52 | 0.04 | 0.02 | 0.02 | 0.06 | 0.02 |

L-leaves; S- stem; R- roots, C- caryopsis

**Supplementary Table 3.** The contribution of individual biochemical and growth parameters to PC1 and PC2 [%]

|  | Contribution [%] | |
| --- | --- | --- |
| **Biochemical or growth parameters** | PC1 | PC2 |
| **CAT** | 12.34514 | 1.375484 |
| **SOD** | 5.614616 | 16.82229 |
| **POX** | 12.26855 | 8.055341 |
| **APX** | 14.03461 | 5.854754 |
| **H_2_O_2_** | 0.002599 | 11.65415 |
| **MDA** | 12.5625 | 8.478893 |
| **GSH** | 4.203117 | 12.1508 |
| **tASC** | 11.75398 | 8.023095 |
| **ASC** | 10.53573 | 4.39781 |
| **ASCr** | 0.135178 | 9.516119 |
| **FW** | 5.559772 | 12.88525 |
| **DW** | 10.98421 | 0.78602 |

APX: ascorbate peroxidase; ASC: ascorbate; ASCr: reduced ascorbate; DW: dry weight; FW: fresh weight; GSH: glutathione; H_2_O_2_: hydrogen peroxide; MDA: malondialdehyde; POX: peroxidase; SOD: superoxide dismutase; tASC: total ascorbate.
